# Encapsulation of Cadmium Selenide Nanocrystals in Biocompatible Nanotubes: DFT Calculations, X‐ray Diffraction Investigations, and Confocal Fluorescence Imaging

**DOI:** 10.1002/open.201700184

**Published:** 2018-01-18

**Authors:** David G. Calatayud, Haobo Ge, Navaratnarajah Kuganathan, Vincenzo Mirabello, Robert M. J. Jacobs, Nicholas H. Rees, Craig T. Stoppiello, Andrei N. Khlobystov, Rex M. Tyrrell, Enrico Da Como, Sofia I. Pascu

**Affiliations:** ^1^ Department of Chemistry University of Bath, Claverton Down Bath BA2 7AY UK; ^2^ Department of Electroceramics Instituto de Ceramica y Vidrio—CSIC Kelsen 5, Campus de Cantoblanco 28049 Madrid Spain; ^3^ Department of Materials Imperial College London, Exhibition Road SW7 2AZ London UK; ^4^ Department of Chemistry, Chemistry Research Laboratory University of Oxford Mansfield Road Oxford OX1 3TA UK; ^5^ Nanoscale & Microscale Research Centre (nmRC), Faculty of Science, School of Chemistry University of Nottingham University Park Nottingham NG7 2RD UK; ^6^ Department of Pharmacy and Pharmacology University of Bath, Claverton Down Bath BA2 7AY UK; ^7^ Department of Physics University of Bath, Claverton Down Bath BA2 7AY UK

**Keywords:** beta-d-glucan encapsulation, cadmium selenide functional nanohybrids, density functional calculations, functional and fluorescent biocompatible nanotube hybrids, self-assembly

## Abstract

The encapsulation of CdSe nanocrystals within single‐walled carbon nanotube (SWNT) cavities of varying dimensions at elevated temperatures under strictly air‐tight conditions is described for the first time. The structures of CdSe nanocrystals under confinement inside SWNTs was established in a comprehensive study, combining both experimental and DFT theoretical investigations. The calculated binding energies show that all considered polymorphs [(3:3), (4:4), and (4:2)] may be obtained experimentally. The most thermodynamically stable structure (3:3) is directly compared to the experimentally observed CdSe structures inside carbon nanotubes. The gas‐phase DFT‐calculated energy difference between “free” 3:3 and 4:2 structures (whereby 3:3 models a novel tubular structure in which both Cd and Se form three coordination, as observed experimentally for HgTe inside SWNT, and 4:2 is a motif derived from the hexagonal CuI bulk structure in which both Cd and Se form 4 or 2 coordination) is surprisingly small, only 0.06 eV per formula unit. X‐ray powder diffraction, Raman spectroscopy, high‐resolution transmission electron microscopy, and energy‐dispersive X‐ray analyses led to the full characterization of the SWNTs filled with the CdSe nanocrystals, shedding light on the composition, structure, and electronic interactions of the new nanohybrid materials on an atomic level. A new emerging hybrid nanomaterial, simultaneously filled and beta‐d‐glucan coated, was obtained by using pristine nanotubes and bulk CdSe powder as starting materials. This displayed fluorescence in water dispersions and unexpected biocompatibility was found to be mediated by beta‐d‐glucan (a biopolymer extracted from barley) with respect to that of the individual inorganic material components. For the first time, such supramolecular nanostructures are investigated by life‐science techniques applied to functional nanomaterial characterization, opening the door for future nano‐biotechnological applications.

## 1 Introduction

Single‐walled carbon nanotubes (SWNTs) have unique structural and electronic properties, which render them very promising materials for device fabrication. In this sense, the insertion and subsequent characterization of a one‐dimensional crystal formed within SWNTs^1^ have attracted a great deal of attention. However, to the best of our knowledge, CdSe nanocrystals have not yet been encapsulated within these strands, and the potential applications of CdSe@SWNTs as functional nanomaterials have not yet been explored. In this context, the synthesis of inorganic nanocrystals encapsulated in SWNTs has been considered as a possible route for studying the properties and applications of low‐dimensional materials. SWNTs have been filled with a variety of materials including transition‐metal halides and chalcogenides, and the changes in the local chemistry of nanotube‐incorporated crystals have been observed directly by high‐resolution transmission electron microscopy (HRTEM). It is believed that encapsulation of these salts introduces a change in the structure of the included material relative to its bulk form, owing to the reduced space and interactions with the walls of the SWNTs. For example, an earlier structural analysis of HgTe@SWNT showed that coordination of Hg and Te was altered significantly from the tetrahedral coordination found in the bulk HgTe zinc blende structure to trigonal planar and trigonal pyramidal geometries, respectively, in a SWNT composite.1g, 1j In some cases, the encapsulation of 1D crystals (e.g. KI) in SWNTs yields a structure without an overall change, but with a systematic reduction of coordination mode.1e In HRTEM, heavy atoms can be observed at higher resolution; however, the positions of light atoms are a challenge, so theoretical model structures must be proposed to generate images for comparison with experimental data. In this sense, first principles calculations have been supportive in predicting the structures of nanocrystals found inside the nanotube and have the bonus of elucidating physical and chemical properties of the composites. Although there is a substantial computational cost associated with studying such large systems, few attempts have been made to study the structures of 1D crystals encapsulated within SWNTs. In this regard, Kuganathan and Green have shown theoretically that HgTe structures inside SWNTs, proposed as a result of the experiment, are in excellent agreement with the detailed theoretical calculations.^2^ Furthermore, gas‐phase density functional theory (DFT) calculations reproducing the experimental results on KI@SWNT are available in the literature.^3^

In this work, we have filled nanotubes of two different SWNT batches (arc‐made and CVD‐made, for comparison purposes) with CdSe, for the first time, and completely characterized the CdSe@SWNTs hybrids emerging from the filling by X‐ray powder diffraction, Raman spectroscopy, HRTEM, and energy‐dispersive X‐ray spectroscopy (EDX) analyses. We have also isolated and characterized (in bulk) an intermediate hybrid material, denoted as CdSe[CdSe@SWNT], which comprises simultaneously filled and coated nanotubes, and shows dispersibility in water and luminescence in the blue‐to‐green region of the light spectrum. These results pave the way for the formation of new luminescent nanohybrids that retain their functionality in aqueous dispersions. Theoretical calculations based on DFT have been employed to elucidate the nature of the thermodynamically stable 1D CdSe nanocrystals that can be formed inside the SWNTs, and an evaluation of the usefulness of this material for sustainable chemistry applications was carried out by using techniques at the interface between life sciences and nanomaterials research.

We hereby report a comprehensive structural study of new functional nanocrystalline materials formed as a result of self‐ and directed‐assembly processes under confinement from bulk materials in the presence of a relatively under‐studied biopolymer, the polysaccharide β‐d‐glucan. This aims at understanding the nature of the CdSe crystals when confined within the SWNT cavities, the impact on their likely optical properties, and considerations of the biocompatibility mediated by this glucan.^3^

## 2 Results and Discussion

### 2.1 Hybrid Nanoassembly Methodology

**Figure 1:**
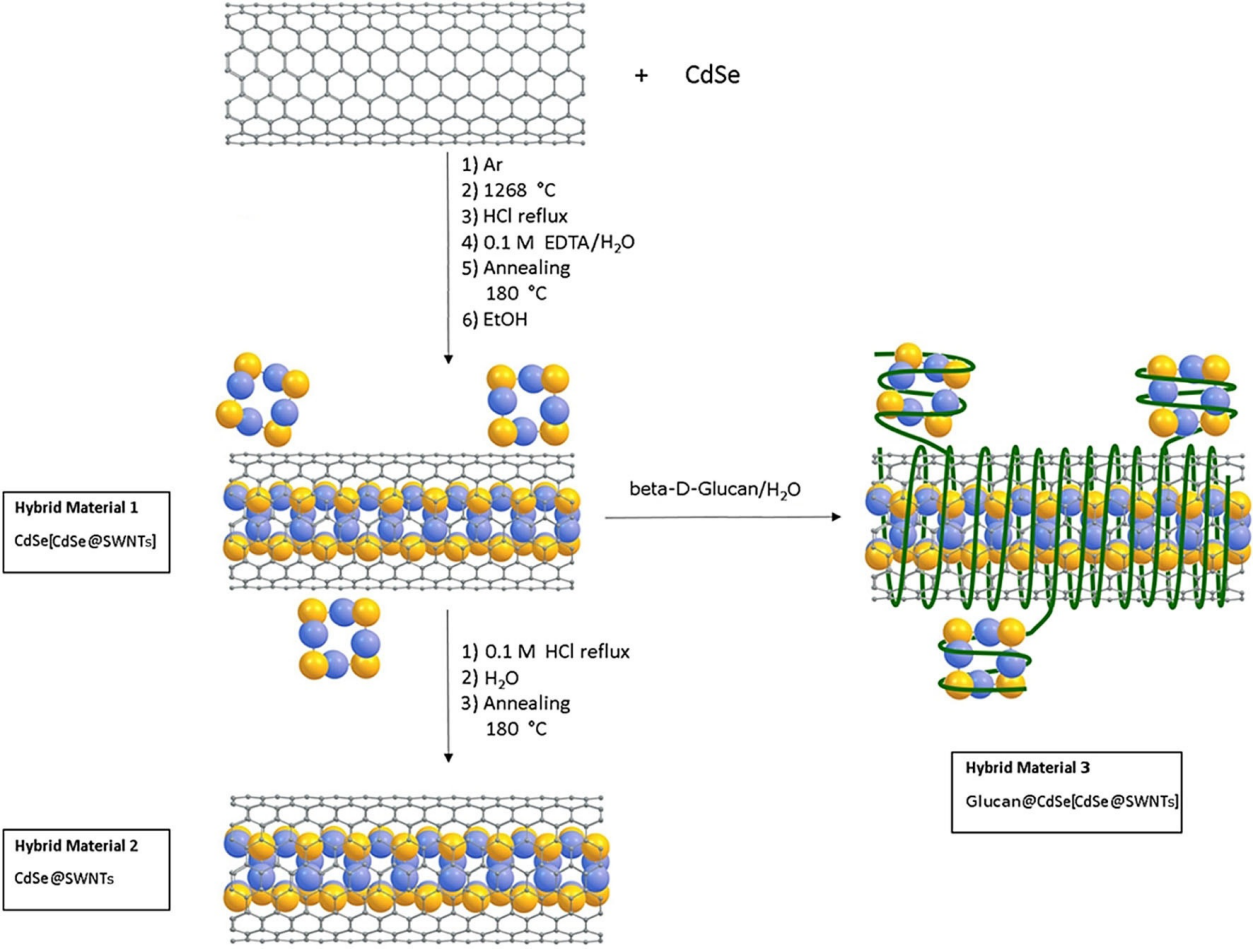
Schematic diagram of the formation and purification of CdSe@SWNTs (Cd: blue; Se: yellow).

Two different batches of SWNTs [electric arc growth (Arc‐SWNT) and chemical vapor deposition (CVD‐SWNT)] were used for comparison purposes. Both SWNT samples were filled with CdSe nanocrystals and purified following the method described by Salzmann and co‐workers^4^ as well as Green and co‐workers^5^ (specific details are given in the Experimental Section). All results were consistent with those previously reported.^5^, ^6^ In the synthetic approach (Figure 1), an annealing process involving temperatures approximately 50 °C above the melting point of the CdSe starting material was used, with the cautious exclusion of air and moisture, to fill the SWNTs. In both cases, hybrid material **1** was isolated and showed similar features in SEM investigations.

**Figure 2:**
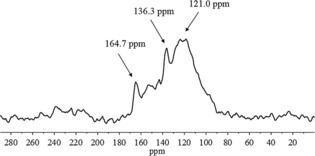
Solid‐state MAS ^13^C NMR (298 K, 10000 Hz) spectrum of pristine CVD‐made and steam purified SWNTs.

Steam‐purified CVD (Elicarb©, Thomas Swann) pristine SWNTs with open tips were thoroughly characterized in bulk by using Raman and solid‐state ^13^C NMR spectroscopy to extrapolate the diameter distribution of the starting material (Figure 2). To ensure the advanced purity of the material used, and its comparable features to previous batches used in similar high‐temperature‐based solid‐state filling methods, the pristine CVD and Arc‐made SWNTs were firstly fully characterized by solid‐state ^13^C NMR, Raman spectroscopy, and HRTEM. The diameter and number of concentric walls of carbon nanotubes (CNTs) can be extrapolated from solid‐state ^13^C NMR by analyzing the chemical shifts of carbon NMR resonances.^7^ In this context, the solid‐state ^13^C NMR spectrum of pristine CVD‐made and steam‐purified SWNTs was de‐convoluted into three overlapping Gaussian peaks (*δ*=121.0, 136.3, and 164.7 ppm), following an approach analogous to that proposed by Engtrakul, Blackburn and collaborators.7b The peak centered at 121 ppm, which represents 91.5 % of the whole sample, shows a chemical shift consistent with those (between 118.8 and 123.8 ppm) reported for SWNTs.^7^ The average diameter of the SWNTs was estimated to be 0.99±0.20 nm. The resonances at 136.3 and 164.7 ppm may indicate the presence of impurities, structural defects, or the presence of C−O bonds, and are equivalent to 4.5 and 4.0 % of the whole sample, respectively.

The filling of SWNTs with metal ions through a solid‐state molten‐salt approach using CdSe requires meticulous removal of air and moisture overnight on a Schlenk line followed by a rather elevated‐temperature insertion, and was performed following the established method by Sloan et al.1e The Elicarb SWNTs samples of advanced purity were then used for the larger scale (5 mg) crystallization of CdSe nanocrystals inside SWNTs. For comparison, the same thermal treatment was applied to the SWNT‐free bulk CdSe material. During the thermal treatment (*T* ª >1268 °C, under argon, with strictly controlled ambient environment during filling, using standard Schenk line techniques), the bulk cadmium selenide was converted into a nanocrystalline material encapsulated in the inner channels of SWNTs, presumably owing to capillary wetting in the molten phase that then allowed crystallization upon controlled overnight cooling in the constraining presence of SWNTs of varying diameters. Arc‐made SWNTs (of Carbolex©, Rice University variety, and further steam‐purified) were treated following the same procedure in order to fill them with CdSe.

To clean the surface of the obtained samples and remove the CdSe particles adsorbed to the external surface of the tubes, the hybrids were cleaned by applying an acid treatment with HCl under reflux, and then they were washed with a 0.1 m EDTA/H_2_O solution and finally annealed at 180 °C. The obtained products were dispersed in absolute ethanol to give the sample denoted hybrid material **1** CdSe[CdSe@SWNT] or in water, in the presence of β‐d‐glucan, for advanced characterization and evaluation of their functionality, hybrid material **3**. Alternatively, in an advanced purification process, hybrid material **1** was treated with reflux in 0.1 m aqueous HCl followed by washing with Milli‐Q water and annealing, leading to the sample CdSe@SWNT, denoted hybrid material **2**. The HRTEM estimated that the filling yield was in the range of 70 % (a statistical treatment of several TEM and HRTEM images was carried out to have an analysis as real as possible of the sample as the bulk).

### 2.2 Nanohybrid Characterization in the Solid State and Dispersed Phase

**Figure 3:**
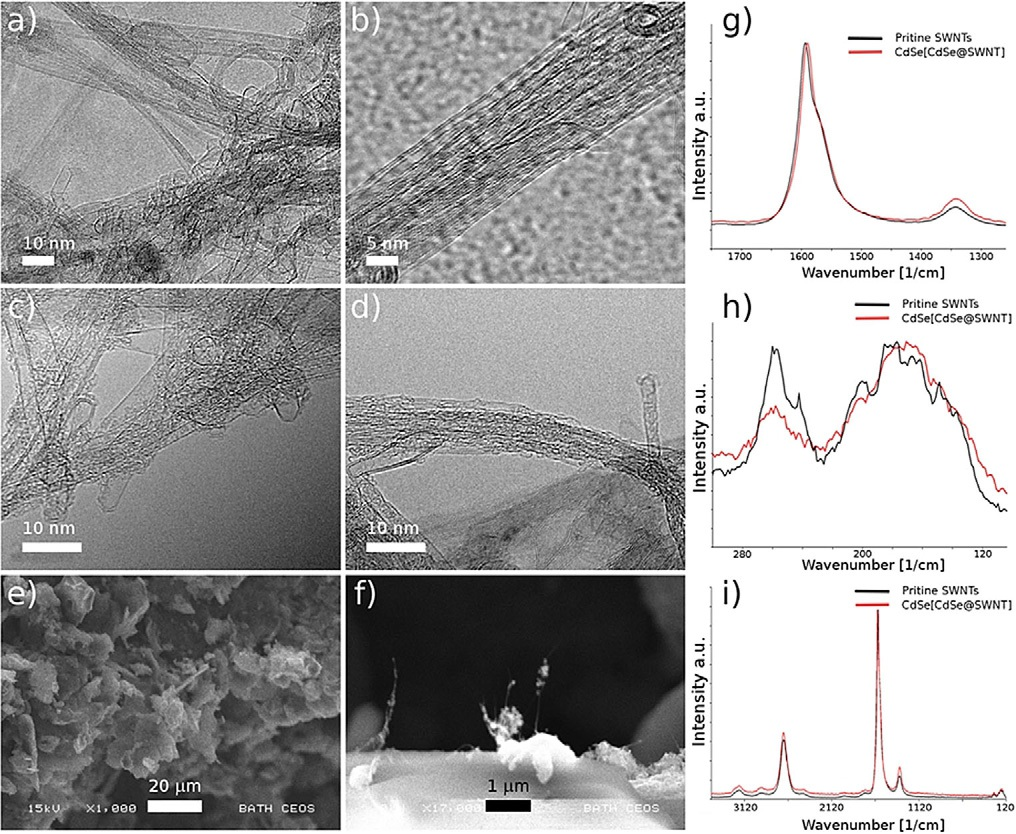
a–d) TEM and HRTEM images; e–f) SEM images of the CdSe[SWNT@CdSe] (hybrid material **1)**, isolated at step 1. g–i) Raman solid state of CdSe[CdSe@SWNT] hybrid material **1** and the pristine CVD‐SWNTs.

As described, CdSe‐filled SWNTs were obtained by mixing the SWNTs with the excess filling material in a silica ampoule sealed under vacuum in two‐stage processes. This resulted in the formation of a nanohybrid denoted as hybrid material **1** (CdSe[CdSe@SWNT], Figure 1), which was surprisingly stable with respect to loss of inorganic materials coating even after a further mild washing with diluted HCl (0.1 m) and H_2_O followed by filtration. This hybrid material **1** was characterized by TEM, SEM, and solid‐state Raman spectroscopy and compared with pristine SWNTs (Figure 3).

**Figure 4:**
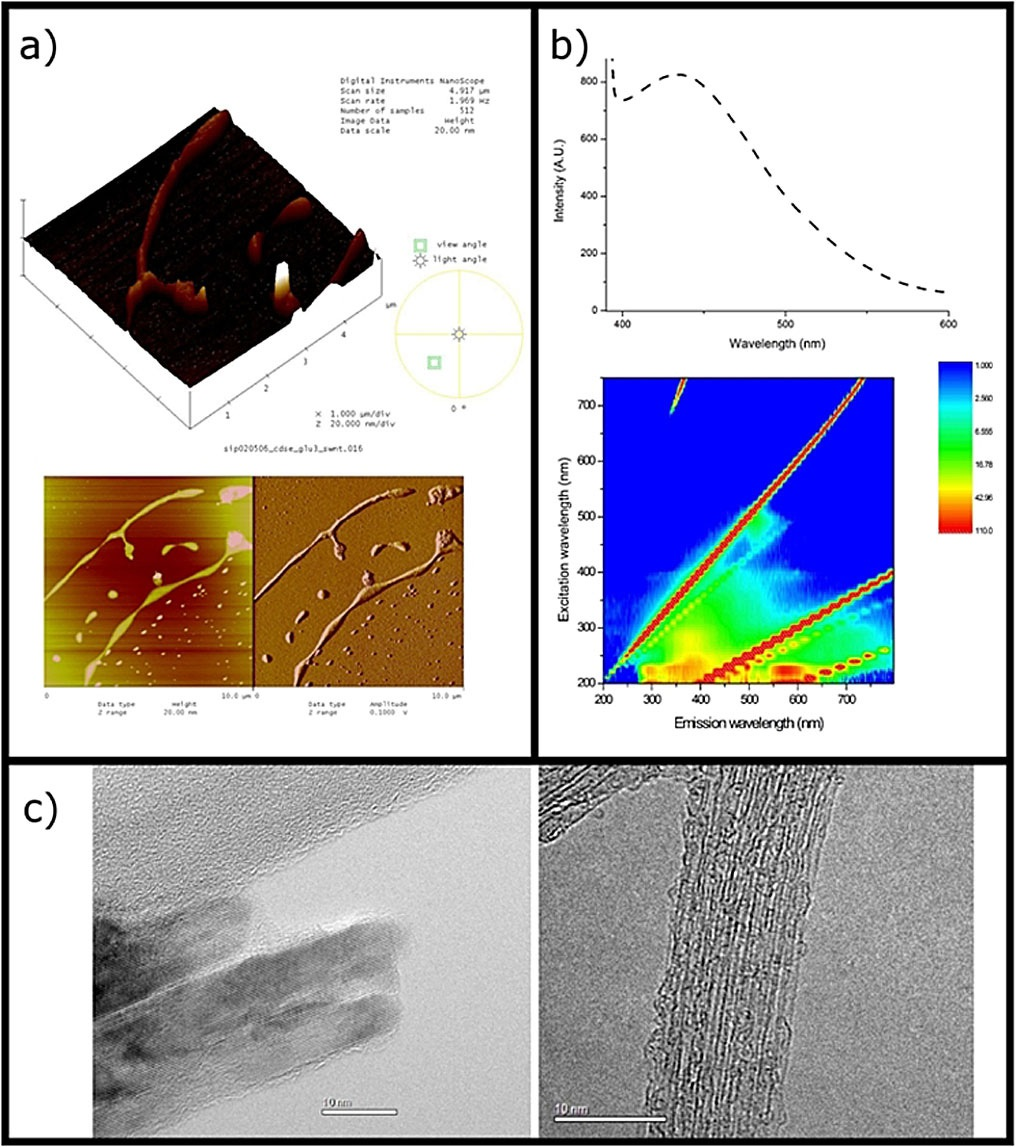
a) AFM of hybrid material **3** in β‐d‐glucan dispersion. b) Fluorescence spectra of hybrid material **1** in 20 mg dispersions using 3 mL of glucan solution, 1 mg mL^−1^ conc in H_2_O (ex 350 nm) and complete excitation‐emission fluorescence map of this dispersion. c) TEM images of annealed CdSe crystals subsequently dispersed in water and using beta‐d‐glucan as a dispersant media (left) and hybrid material **3** in β‐d‐glucan (1 mg mL^−1^) dispersion (right).

Furthermore, beta‐1,3‐1,4, glucan from barley, also known as β‐d‐glucan (i.e. a water‐soluble derivative of a well‐known glucan‐based dispersants for SWNTs,^9^ which is also known to form a single‐stranded polymeric chain in DMSO and self‐assemble as a triple helix in water) was used as the adjuvant of choice to aid the formation of an aqueous dispersion of hybrid material **1**, improving its solubility in water, leading to hybrid material **3** (Figure 1). This was fully characterized and an additional functionality was imparted by glucan coating (Figure 4). The obtained glucan‐wrapped material was re‐dispersed in H_2_O or H_2_O/DMSO mixtures, giving composites that are stable for weeks in aqueous media. Aggregates of barley beta‐d‐glucan‐coated SWNTs seem to emerge from dispersions after centrifugation, and these were visualized by AFM, TEM, and fluorescence spectroscopy and compared to those of CdSe‐free, SWNTs@glucan hybrids (vide infra). An advanced purification process of hybrid material **1** was carried out, leading to CdSe@SWNT, denoted hybrid material **2**. For hybrid material **2**, only a minimum inorganic extraneous material (CdSe or decomposition products) was found by HRTEM/EDX and XRD in both types of SWNTs (CVD‐SWNTs and Arc‐SWNTs samples).

**Figure 5:**
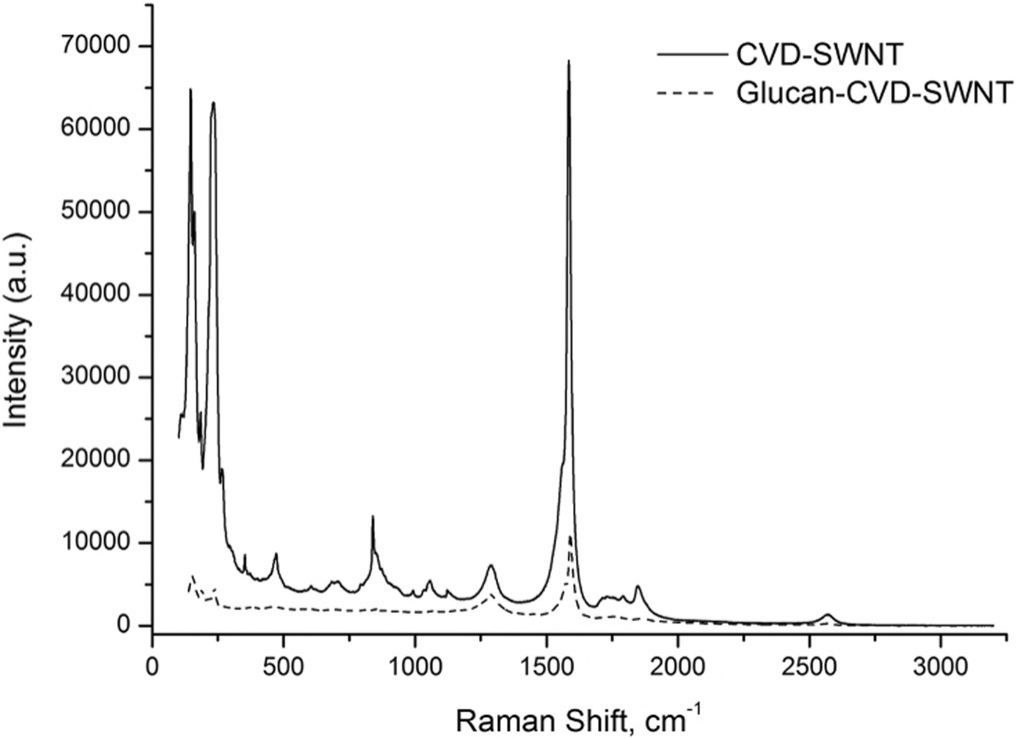
Resonant Raman spectra of CVD‐SWNT and glucan‐CVD‐SWNT at 830 nm.

The presence of a diverse nanotube size distribution in the sample was also observed in dispersions of the hybrid materials in natural biopolymers such as β‐d‐glucan (from barley, Aldrich) diffusion‐oriented spectroscopy (DOSY) experiments on the ^1^H NMR spectrum of β‐d‐glucan‐wrapped CdSe‐ [CdSe@SWNT], hybrid material **3**. Figure S20 (see the Supporting Information) shows the DOSY ^1^H NMR spectrum of hybrid material **3** in a 1:1 D_2_O:[D_6_]DMSO solution, within the region of the spectrum associated with the signals of glucan; six NMR spin systems can be observed, with values of diffusion coefficients between 1.61 ×10^−10^ ±1.06 ×10^−11^ and 7.90 ×10^−12^ ±1.04 ×10^−11^ m^2^ s^−1^. Therefore, it can be concluded that, when in solution, the fibers of β‐d‐glucan wrap the SWNTs and generate nanohybrid species of different sizes, which are soluble in D_2_O and [D_6_]DMSO. Raman spectroscopy (830 nm laser) measurements of CVD‐SWNTs and dispersed CVD‐SWNT (in presence of β‐d‐glucan) were carried out to estimate the density of structural defects in CNTs. The intensity ratio of the Raman d band to g band (*I*_D_/*I*_G_) provides a relative measure of the density of structural defects and, therefore, the structural quality of a sample.^8^ If both of these bands are similar in intensity, the density of structural defects is assumed to be high. Figure 5 shows the Raman spectra for CVD‐SWNT and glucan‐CVD‐SWNT samples. It can be observed that the starting material presents a D band at 1288 cm^−1^ and a G band at 1584 cm^−1^.

The *I*_D_/*I*_G_ ratio of the CVD‐SWNT material, reported in Table 1, was found to be 0.107, indicating a low level of surface defects and disorder.^8^ The sharp D and G bands also confirm that the provided tubes are single‐walled rather than multi‐walled in structure. The CVD‐SWNT also exhibits three sharp peaks below 300 cm^−1^ (146, 160, and 233 cm^−1^), which correspond to the radial breathing mode (RBM). The calculated diameters from these RBM bands are 1.62, 1.50, and 0.85 nm, respectively. The other peaks observed between 300 and 1300 cm^−1^ are probably due to residual metal nanoparticle catalyst impurities (majorly Fe residual). It is also expected that they are wrapped in carbonaceous material. On the other hand, the Raman spectrum for the glucan‐CVD‐SWNTs shows a shift in the G band to higher wavenumber (1590 cm^−1^), owing to the presence of glucan on the surface. The D band appears at the same position (1288 cm^−1^). The *I*_D_/*I*_G_ ratio increased to 0.342, which can be attributed to the presence of glucan adsorbed on the surface and/or an increased on the level of surface defects and disorder. However, the value is still low, indicating a good structural quality. The peaks corresponding to the RBM are also shifted with respect to the CVD‐SWNTs (152, 189, 237 cm^−1^), owing to wrapping with glucan.

**Table 1:** Intensities and intensity ratio of the Raman d band to G band of the CVD‐SWNTs and glucan‐CVD‐SWNTs.

Sample^[a]^	D‐band absorbance [cm^−1^]	G‐band absorbance [cm^−1^]	*I*_D_/*I*_G_
Purified SWNTs	7322	68266	0.107
Dispersed SWNTs	3828	11190	0.342

[a] In the presence of glucan.

**Figure 6:**
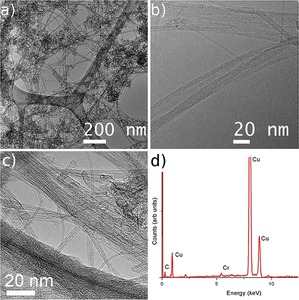
TEM and HRTEM imaging of CVD‐SWNTs (steam‐purified at 900 °C, 2 h): a) lower magnification image; b, c) higher magnification images of SWNTs. d) EXD analysis (Cu lacey grids, dispersed from EtOH). Images show high‐purity SWNTs as well as the presence of some MWNTs and DWNTs, with traces of amorphous material.

The morphology and structure of CVD‐SWNTs were investigated by HRTEM. Figure 6 shows the microscopy images of the ultrapure CVD‐SWNTs, which confirm good structural quality with clean surfaces. The SWNT diameters are in agreement with those estimated from Raman and ^13^C NMR data.

**Figure 7:**
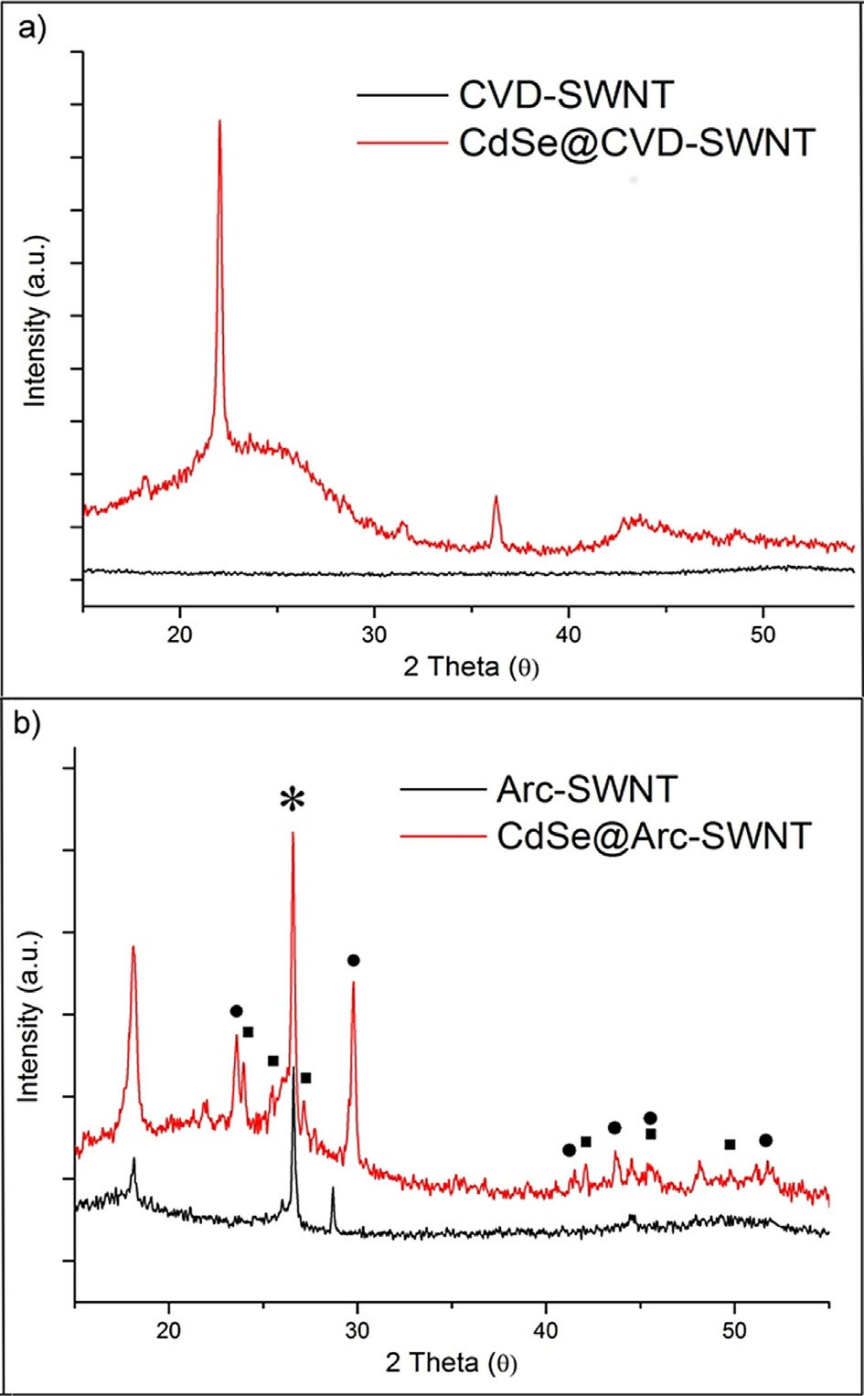
Powder X‐ray diffraction patterns of a) pure CVD‐SWNT and the composite CdSe@CVD‐SWNT and b) pure Arc‐SWNT and CdSe@Arc‐SWNT. * refers to the nanotubes; ▪ refers to the Cadmoselite CdSe phase; peaks labeled with • are assigned to hexagonal selenium phase. The peaks without a mark correspond to different defects on the SWNTs and/or carbonaceous species sub‐products.

The grade of crystallinity of pristine SWNTs and purified CdSe@SWNTs (hybrid material **2**) was further investigated by X‐ray diffraction (Figure 7). As inferred from the pattern depicted in Figure 7 a, the CVD‐SWNT sample is not crystalline, whereas the SWNTs prepared by electric arc growth present some crystallinity, as confirmed by the Bragg peaks in the spectrum (Figure 7 b). XRD analysis of the CVD‐SWNT sample after the thermal treatment with CdSe[CdSe@CVD‐SWNT] shows Bragg peaks that can be attributed to carbonaceous subspecies and/or surface defects, thereby indicating the partial degradation of the SWNTs under thermal treatment. The Arc‐SWNT sample after the thermal treatment with CdSe (CdSe@Arc‐SWNT) also shows peaks corresponding to carbonaceous subspecies or surface defects, but the pattern also shows the existence of a hexagonal CdSe phase (Cadmoselite, ICDD 08–0459) together with a hexagonal selenium phase (ICDD 06–0362).

**Figure 8:**
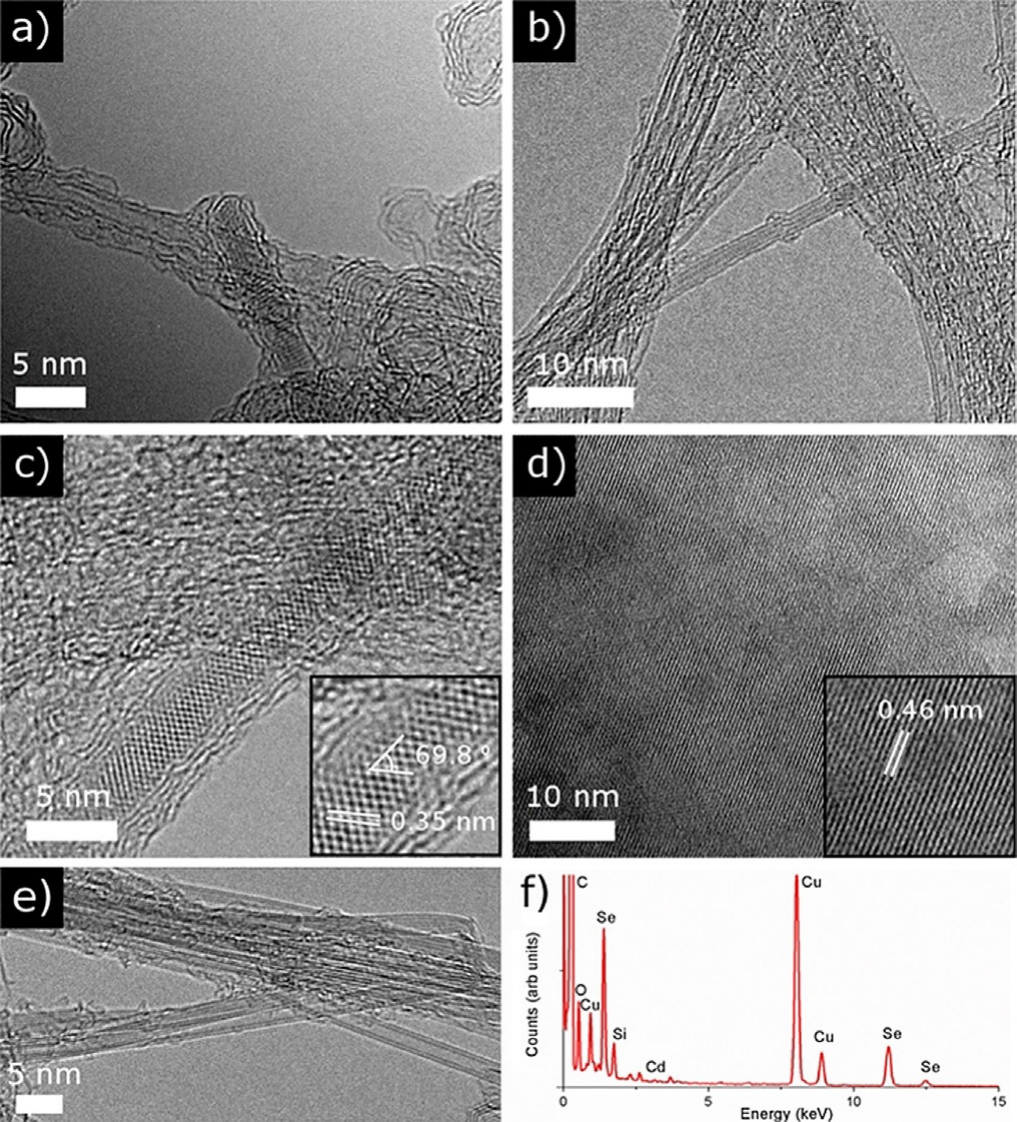
HRTEM images of the advanced‐purified CdSe@SWNTs samples denoted as hybrid material **2**: a) CVD‐SWNT sample filled with CdSe (CdSe@CVD‐SWNT); b) Arc‐SWNT sample filled with CdSe (CdSe@Arc‐SWNT); c) higher magnification of an individual Arc‐SWNT filled with CdSe (the inset shows the interplanar crystal spacing and the interfacial angle); d) a hexagonal CdSe particle (the inset shows the interplanar crystal spacing); e) CdSe@CVD‐SWNTs confirming that a mixture of SWNTs, DWNTs, and MWNTs is present, where SWNTs have a diameter of approximately 1.7 nm; f) corresponding EDX, which shows the presence of Cd and Se for the CVD‐SWNT sample filled with CdSe (CdSe@CVD‐SWNT).

TEM images of the CdSe@SWNTs (hybrid material **2**) show structural fragments of intercalated material in both samples, but to a wider extent in the CdSe@Arc‐SWNT sample (Figures 8 a and 8 b). So, it is confirmed that, during the thermal treatment, the CdSe melt was encapsulated in the inner channels of SWNTs, owing to capillary wetting. The EDX analyses confirm the presence of both Cd and Se in the CdSe@CVD‐SWNT sample.

After careful scrutiny of the HRTEM images at several different magnifications (see the Supporting Information), there is no evidence of CdSe or Se aggregates outside the NTs. Close examination of the encapsulated crystal by electron diffraction completed with HRTEM in the CdSe@Arc‐SWNT (Figure 8 c) shows that dark spots can form a unit with the shape of a hexagon, and this unit is regularly repeated along the tube axis. Interestingly, this hexagonal pattern can be associated with the crystal phase of the CdSe (Figure 8 d) filling inside the tubes, which is in agreement with the hexagonal CdSe phase observed by X‐ray powder diffraction. The spots observed across the SWNT capillary are spaced at average intervals of 0.59 nm, and along the SWNT capillary the spacing is 0.35 nm with an interfacial angle of 69.8°. This lower interplanar crystal phase of the CdSe filling the SWNTs in comparison with the spacing for a free hexagonal CdSe particles (0.46 nm) (Figure 8 d) indicates that CdSe inside the nanotube is confined and the structure is compressed in order to adapt to the available internal space, as determined by the nature of the nanotube host employed. The diameter of some of the observed SWNT samples is found to range between 1.4 and 1.7 nm, which is close to the diameter of a typical (9,9) SWNT.

**Figure 9:**
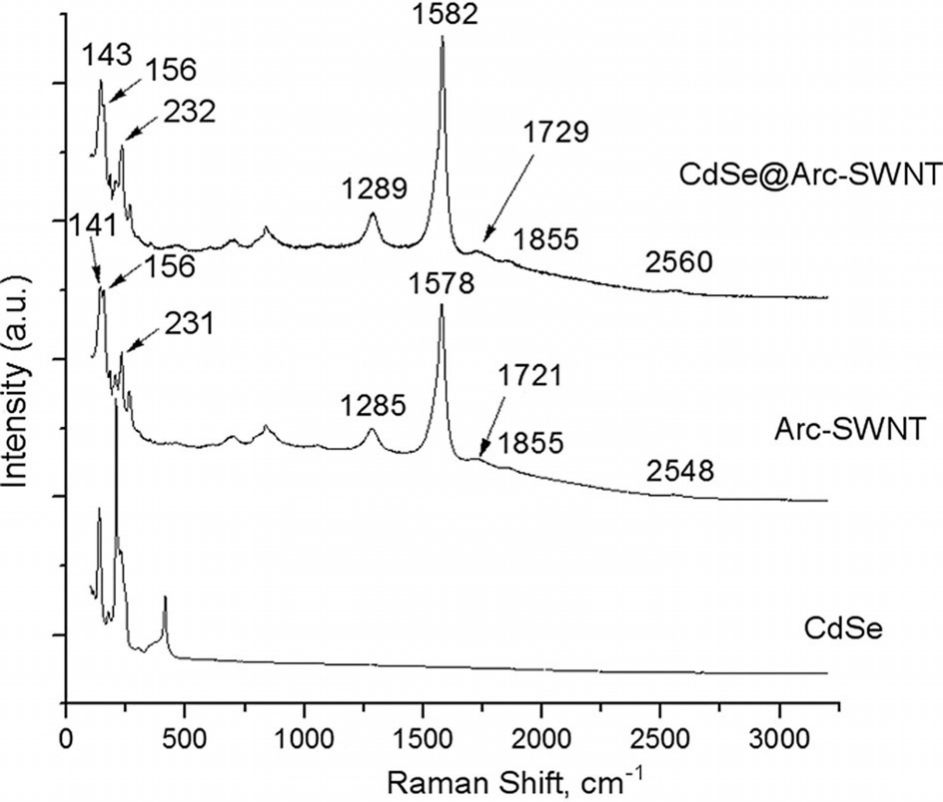
Resonant Raman spectra of CdSe, Arc‐made SWNT, and corresponding CdSe@SWNTs at 830 nm.

Raman spectroscopy measurements allowed the identification of the inner structure of the CdSe@Arc‐SWNT sample (hybrid material **2**), and it shows the optimum achievable filling of the nanotubes with CdSe nanowires under the solid‐state conditions employed (Figure 9). The starting material presents a D band at 1289 cm^−1^ and a G band at 1578 cm^−1^. The *I*_D_/*I*_G_ ratio of this starting material sample of SWNTs was found to be 0.36. Such an *I*_D_/*I*_G_ ratio indicates a low level of surface defects and disorder.^8^ The sharp D and G bands also confirmed that the provided tubes are single‐walled rather than multi‐walled in structure. The starting Arc‐SWNTs also exhibit three sharp peaks below 300 cm^−1^ (at 141, 156, and 231 cm^−1^), which correspond to the RBM. The calculated diameters from the RBM bands are 1.59, 1.43, and 1.01 nm, respectively. The Raman spectrum of intact SWNTs also exhibited several peaks from 300 to 1300 cm^−1^, which were probably caused by the residual metal nanoparticle catalyst impurities (mainly Fe and Cr traces from the catalytic process). It is also expected that they are wrapped in carbonaceous material as suggested by HRTEM.

The *I*_D_/*I*_G_ ratio of SWNTs after thermal treatment was slightly reduced to 0.33, and this decrease can be assigned to the reduction of surface defects (Figure 9). Only the bands assigned to SWNTs were found in the Raman spectrum of SWNTs filled with CdSe (Figure 9). However, Raman bands have undergone a shift to higher frequency. It is known9b–9d that a high‐frequency shift of Raman bands of filled SWNTs takes place if the encapsulated material is an acceptor. The observation of this shift in the spectrum of CdSe@Arc‐SWNT is probably due to the charge transfer from SWNTs to CdSe. The Raman spectrum of CdSe@Arc‐SWNT remained almost unchanged with respect to the Arc‐SWNTs and exhibited three RMB peaks with maxima at 232, 156, and 143 cm^−1^. This suggests the formation of purified tubes with calculated diameters of mainly 1.01, 1.43, and 1.62 nm, respectively, and rather narrow distribution ranges.

### 2.3 Luminescence Studies in the Dispersed Phase for Functional Nanohybrids

There is an increasing demand for water‐soluble in vitro and in vivo molecular probes for imaging and early detection of cancers, and we demonstrate that water‐solubilized β‐d‐glucan carbon nanotubes have an acceptable level of biocompatibility to enable the evaluation of their cellular uptake. The newly synthesized nanohybrids showed biocompatibility comparable with that of the free glucan, both in healthy and in cancerous cells. Luminescent nanowires obtained under extreme conditions display, for the first time, CdSe‐decorated (inside as well as outside, depending on the conditions employed) carbon nanotube strands. These can be “recognized” and individually modified by the polysaccharide fibers of β‐d‐glucan, which act as adjuvants, allowing the complete full characterization of the encapsulated materials in water media.

To evaluate the potential applications of the obtained hybrid materials, the photoluminescence properties of the hybrid functional materials (hybrids **1**, **2**, and **3**) in aqueous media were investigated and compared to those of CdSe quantum dots and commercially available core–shell CdSe/ZnS nanocrystals (Lumidot^TM^ 480). All of these materials were imaged by laser scanning confocal microscopy on thin films, thereby adapting these techniques available at the life science interfaces to address characterization and functionality probing challenges in nanomaterials.

**Figure 10:**
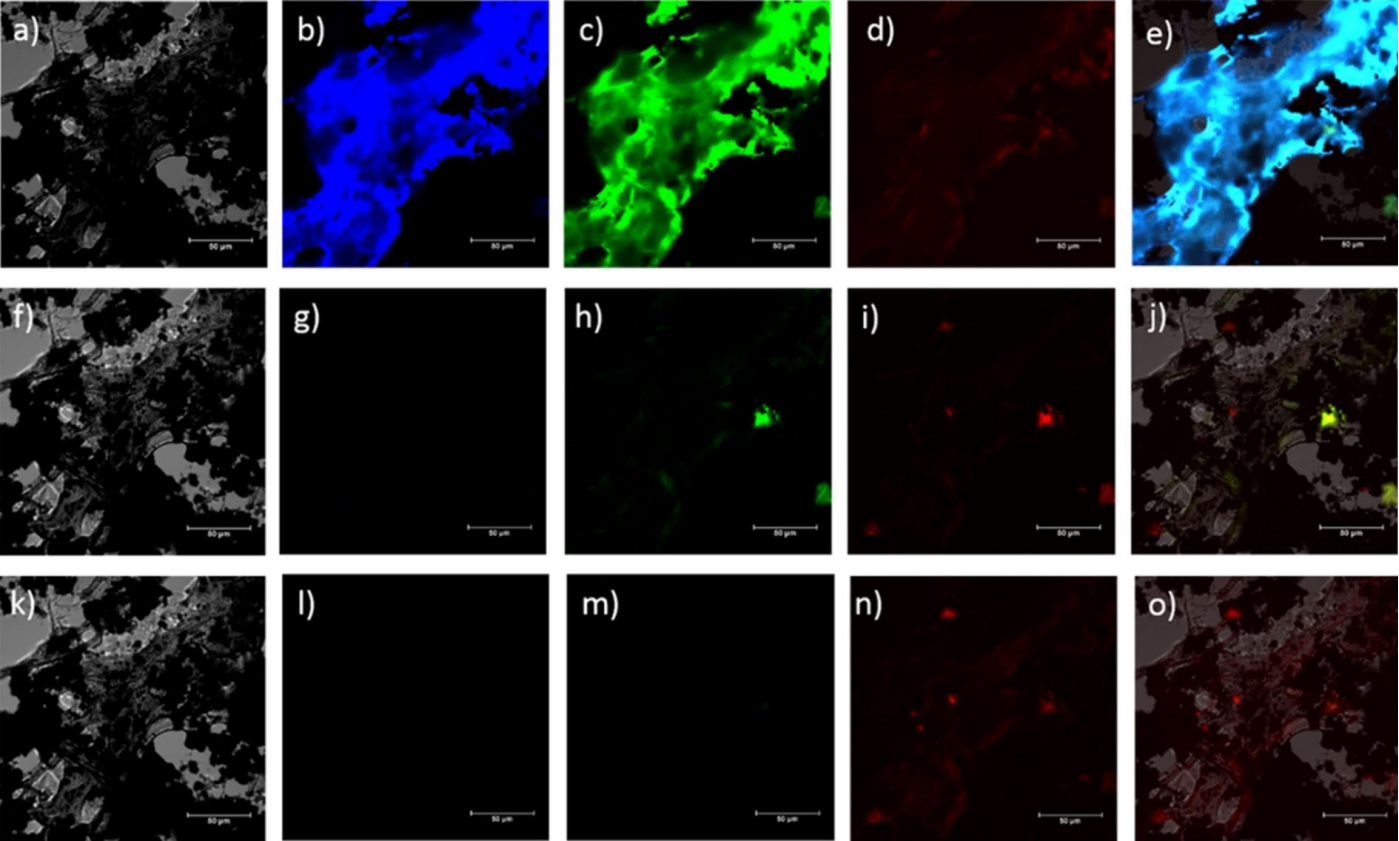
Single‐photon laser‐scanning confocal microscopy of CdSe[CdSe@SWNTs] wrapped in glucan (hybrid material 3). a–e) λ_ex_=405.0 nm; f–j) *λ*_ex_ = 488.0 nm; k–o) *λ*_ex_=561.0 nm. a, f, k) DIC channel; b, g, l) blue channel (*λ*_em_=417–477 nm); c, h, m) green channel (*λ*_em_=500–550 nm); d, i, n) red channel (*λ*_em_=570–750 nm). e, j, o) Overlay of the blue‐green‐red channels. Scale bar: 25 μm.

**Figure 11:**
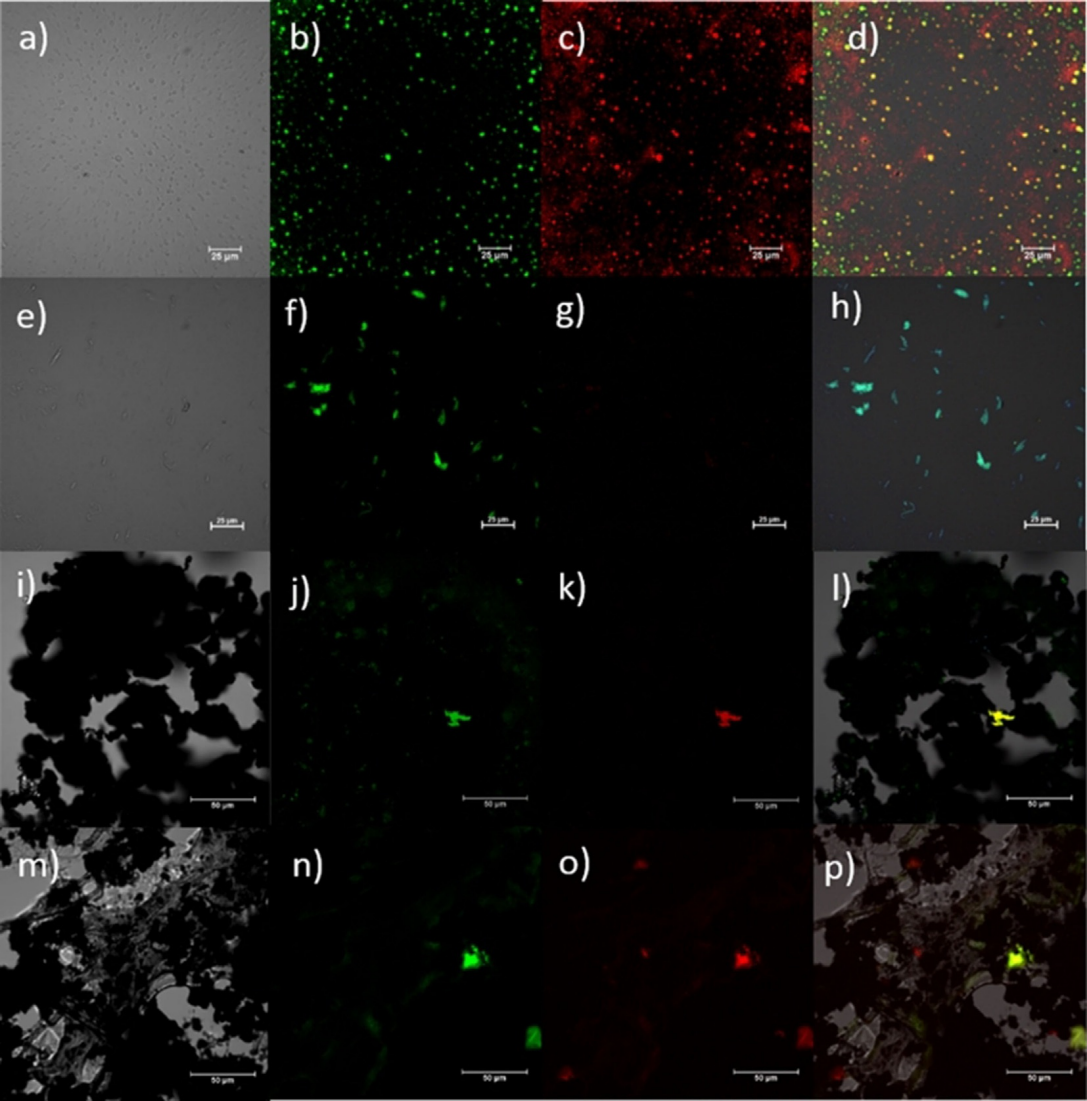
Single‐photon laser‐scanning confocal microscopy (*λ*_ex_=488 nm) of CdSe (a–d), Lumindot^TM^ 480 nm (e–h), CdSe@SWNTs (i–l), and CdSe@SWNTs‐ @glucan (m–p); DIC channel (a, e, i, m); green channel, *λ*_em_=500–550 nm (b, f, j, n); red channel, *λ*_em_=570–750 nm (c, g, k, o); overlay of the channels (d, h, l, p).

Confocal fluorescence microscopy of a dried thin film of water soluble hybrid material **3** was performed, and the corresponding images are shown in Figure 10. The figure shows bright‐field images (Figures 10 a, 10 f, and 10 k), individual channel emissions (blue, Figures 10 b, 10 g, and 10 l: *λ*_em_=417–477 nm; green, Figures 10 c, 10 m, and 3 h: *λ*_em_=500–550 nm; red, Figures 10 d, 10 i, and 10 n: *λ*_em_=570–750 nm), and the emission overlay of the blue–green–red and DIC channels (Figures 10 e, 10 j, and 10 o) at the relevant excitation wavelength (*λ*_ex_=405 nm, Figures 10 a–e; *λ*_ex_=485 nm, Figures 10 f–j; *λ*_ex_=561 nm, Figures 10 k–o). The glucan‐wrapped water dispersible hybrid material **3**, forming a thin film, was found to emit in the blue (417–477 nm) and red (570–750 nm) range when excited with a 405 nm laser light. A direct comparison of such emission performances with those of hybrid material **2** (Figures S5 a–e) seems to suggest that the coating of the CdSe@SWNT with glucan directly affects the absorption/emission properties of the composite material, enhancing its capability to emit at higher frequencies. Interestingly, if we irradiated a thin film of CdSe with a 488 nm excitation laser light, the microscopy images (Figures 11 a–d) reveal a strong fluorescence intensity in both green (*λ*_em_=417–477 nm) and red (*λ*_em_=570–750 nm) channels, and only in green channel for a thin film of the commercially available CdSe Lumindot^TM^ 480 nm. Such emission properties are significantly quenched when CdSe structures are confined within nanotubes or glucan‐nanotubes nanostructures, as seen in Figures 11 i–l and 11 m–p, respectively, suggesting the formation of a photoexcited energy‐transfer complex.^10^

Single‐photon laser‐scanning confocal microscopy of CdSe, in the form of the commercially available Lumindot^TM^ 480 nm and CdSe@SWNTs using 405, 488 and 561 nm laser excitation lines, is reported in the Supporting Information for comparison.

### 2.4 Computational Structural Investigations

As previously mentioned, the diameter of some of the observed SWNTs was found to range between 1.4 and 1.7 nm, which is close to the diameter of a typical (9,9) SWNT. Thus far, structural models are not established for CdSe@SWNTs products and X‐ray diffraction studies coupled with DFT modelling were necessary to shed light on the structure of encapsulated nanocrystals. In a previous theoretical study on CuI@SWNT, we have predicted novel structures for CuI crystals that may be found in SWNTs.^22^ The starting structures of CuI crystals were based on the HgTe structures derived from bulk and observed within SWNTs as the both Cu^+^ and Hg^2+^ have both d^10^ ions. As Cd^2+^ is also a d^10^ ion, it is possible to propose a starting structure for CdSe nanocrystals based on the HgTe or CuI structures available in the literature.1g, 4, 10

Theoretical calculations have been employed in this work for the aid of understanding the structure and stability of CdSe@SWNT hybrid material. Computational parameters used in this study using CASTEP code^11^ (see the Experimental Section) are consistent with those reported in our earlier studies,^12^ where parameters (ultrasoft pseudopotentials generated using the “on the fly” formalism, the cut‐off energy of 320 eV and Monkhorst grid *k*‐points) were checked for convergence, and the results obtained reproduced the experimental observations. The specimens studied hereby showed that a variety of diameters are available for CNT‐templated filling with CdSe, ranging from 1.1 to 3.0 nm for the internal diameters of CNTs, depending on the source of the SWNTs (i.e. CVD, Elicarb, or arc‐made), and were consistent with the HRTEM, Raman, and ^13^C MAS NMR measurements. Figure 8 c shows a selected HRTEM‐imaged CdSe‐filled CVD MWNT strand as an example (whilst additional images of SWNTs and DWNTs are shown in the Supporting Information), whereby a broad CdSe nanowire is encapsulated in a multi‐walled CNT with an inner diameter of at least 3.0 nm. However, the vast majority of the tubes observed showed an average inner diameter of maximum diameter of approximately 1.4 nm. As such, we focused our attention on the theoretical modelling of the tubes with average diameters of 1.4 nm and chose a series of simplified models to investigate further at a computational level.

Three different starting structures are proposed for the CdSe nanocrystal. The first structure is a novel structure related to the HgTe nanocrystal observed within SWNTs (3:3) and the second one is a low‐dimensional structure derived from the NaCl rock salt bulk structure with 4:4 coordination.^2^ The third structure is a model structure related to that predicted for CuI nanocrystals found inside SWNTs (4:2).^13^ The lowest energy structure of these model structures are encapsulated in a (9,9) nanotube based on the experiment and electronic structure and properties are calculated in the following section. First principles DFT calculations were performed on 1D CdSe nanocrystals encapsulated within SWNTs in order to determine the electronic structure and the nature of the interaction between the nanocrystals and SWNTs. The CASTEP code,^11^ which solves the standard Kohn–Sham (KS) equations using plane wave basis sets, was employed in all calculations.

For calculations on infinite CdSe crystals and CdSe@SWNT composites, periodic boundary conditions were applied to enforce a minimum lateral separation of 25 Å between structures in adjacent unit cells. At this separation, the interaction between these structures and their periodic images are negligible.

**Figure 12:**
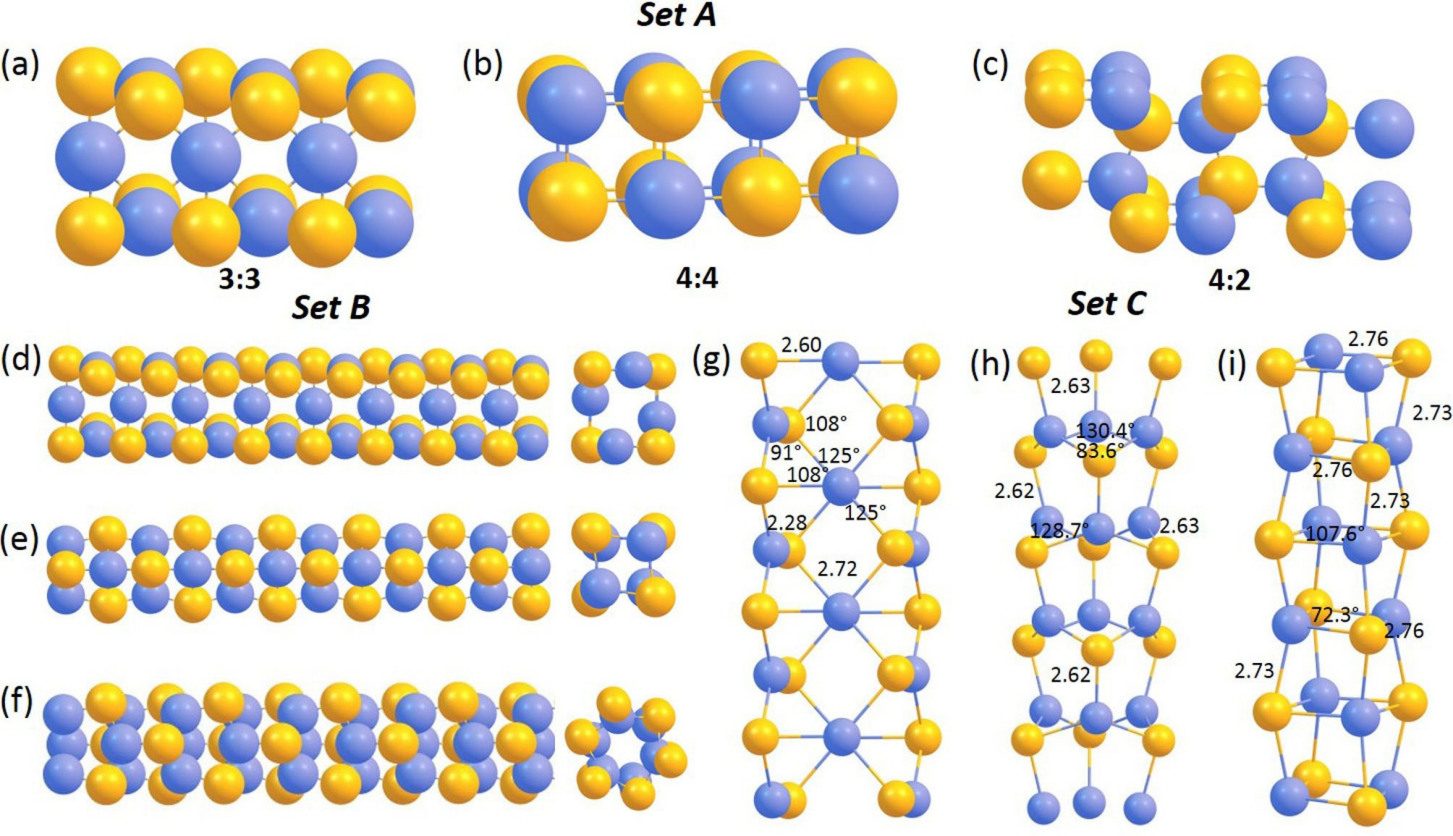
Set A) Three different model 1D crystal fragments of CdSe with 3:3 (a), 4:4 (b), and 4:2 (c) coordination (Cd: blue; Se: yellow), where structures were optimized in the absence of a confining nanotube and using periodic boundary conditions. Set B) The optimized structures of the CdSe fragment of structures 3:3, 4:2, and 4:4, showing different views along or perpendicular the length axes. Set C) Optimized geometries with main geometry parameters of the 1D CdSe nanowires considered, representing the g) 3:3, h) 4:2, and i) 4:4 coordination modes, with periodic boundary conditions set outside the nanotube, also showing bond lengths (Å) and angles (°).

For the DFT modelling of 1D crystals of CdSe, the geometries of three different types of CdSe structures were optimized in the absence of a confining nanotube and using periodic boundary conditions. The initial structures are shown in Figure 12 A, and further optimized modeled structures in the Sets of Figure 12 B and 12 C.

**Figure 13:**
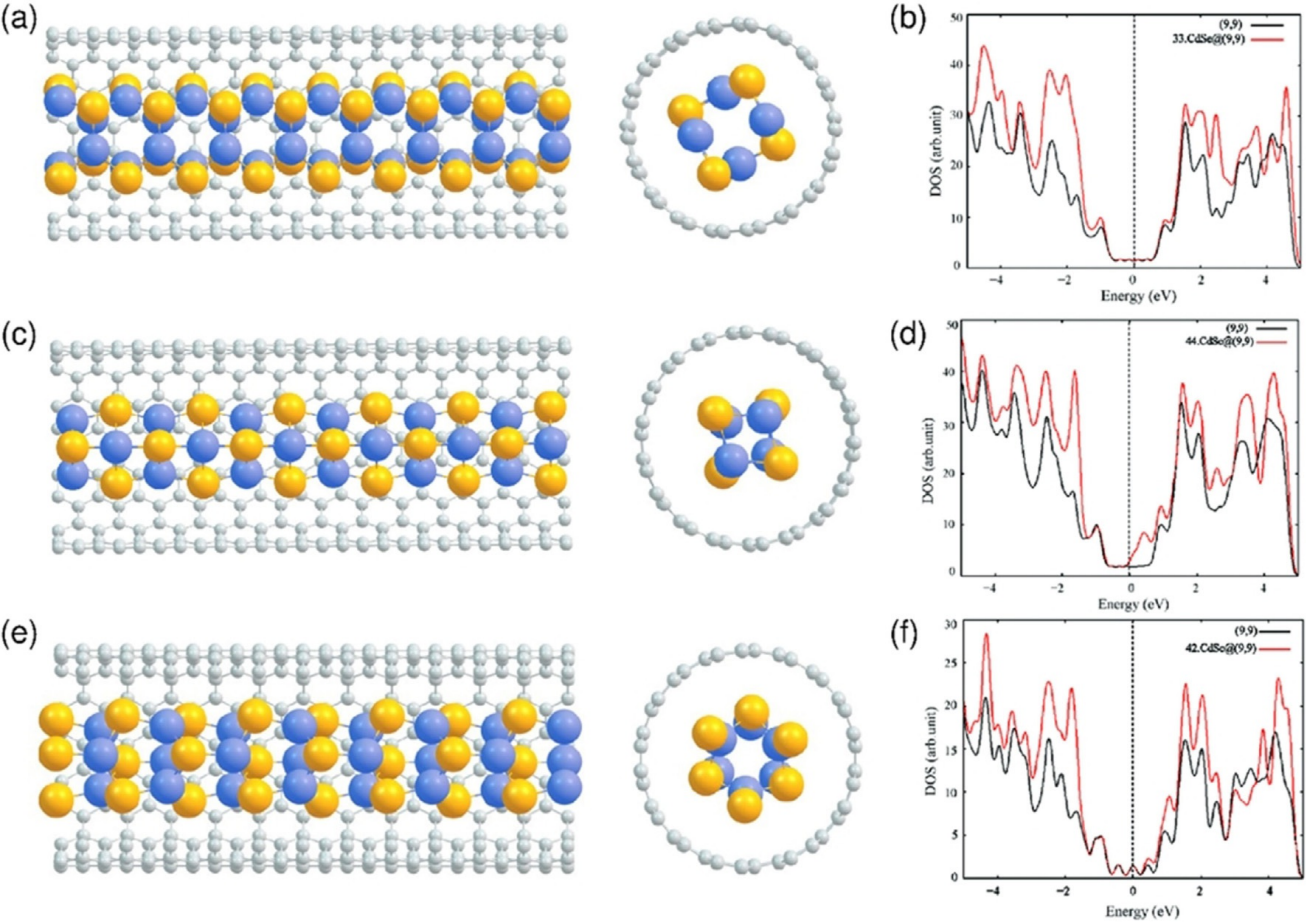
Optimized geometries of a) 3:3, c) 4:4, and e) 4:2 CdSe nanocrystals encapsulated within (9,9) nanotube strands. The corresponding DOS are shown in (b), (d), and (e), respectively, whereby the black lines correspond to the pristine (9,9) tube and the red lines to the tube plus interaction of the CdSe crystal. The marked zero point corresponds to the Fermi energy.

The first modelled structure is directly linked to the experimentally observed novel structure for CdSe within SWNT with 3:3 coordination. In this structure, a Cd atom is in a trigonal planar coordination and Se atom has a pyramidal environment. The second structure is a 2×2 low‐dimensional structure derived from the NaCl rock salt bulk structure with 4:4 coordination of the atoms. The third structure is based on the model structure predicted for CuI structure (derived from the hexagonal CuI bulk structure), which has 4:2 coordination. All three structures were considered as infinite 1D crystals and calculations were performed with periodic boundary conditions. The geometry optimization of these three structures indicates that CdSe (3:3) structure is the lowest in energy. The second most stable structure is 4:2 (higher in energy by 0.06 eV). The relaxed structure of 4:2 forms another three coordinated structure (see Figure 12 C h, which is closer to the 3:3 structure in energy. The optimized structures are shown in Figure 13 and the relative energy per CdSe unit of structures 3:3, 4:2, and 4:4 are given in Table 2.

**Table 2:** Relative energy per CdSe unit of three 1D CdSe crystals (3:3, 4:4, and 4:2).

Polymorph	Relative energy [eV/CdSe]	
3:3	0.00	
4:2	0.06	
4:4	0.25	

The optimized *free* 3:3 structure within periodic boundary conditions gives a structure that resembles the starting structure. However, Cd−Se bond lengths are slightly shorter compared to the starting bond lengths proposed for Hg−Te bonds (2.80 Å). Calculated bond angles are close to the values predicted for HgTe crystals found inside SWNTs. The structural parameters (bond lengths and angles) of the optimized structure of the CdSe crystal are given in Figure 12 C g. Optimization of the 4:2 crystals gave a slightly distorted structure with threefold coordination compared to the initial structure with the Cd−Se bond lengths 2.63 Å, Se−Cd−Se angles close to 130.0°, and Cd−Se−Cd angle 83.6°, as shown in Figure 12 C h, and did not lead to the lowest energy 3:3 structure. The optimized structure of a *free* 4:4 CdSe crystal with periodic boundary conditions gave a rhombohedral structure (Figure 12 C i). The distances of the Cd−Se bonds (2.73–2.76 Å) were longer than the distances found in the 3:3 structure (2.28–2.72 Å). Calculated bond angles Se−Cd−Se and Cd−Se−Cd are found to be 107.6 and 72.3°, respectively.

Model studies focused on a simplified selection based on three different types of polymorphs of CdSe that were encapsulated within a (9,9) nanotube and calculations in the gas phase were performed. The calculated Cd−Se bond lengths, binding energy, and charge transfer are given in Table 3.The calculated binding energies indicate that all three composites can form a CdSe molten state in the experimental approach. The main contribution for binding energy was found to be the van der Waals interactions in such tubes; however, the reasons for the formation of nanowires in a CNT‐templated environment are likely to be kinetic, as well as thermodynamic, in nature. Our calculations of the density of states (DOS) for a typical (9,9)‐SWNT show that filling CdSe nanowires into the nanotube sidewall only very subtly perturbs the electronic structure of the SWNT, likely owing to non‐covalent interactions between the π‐electronic system of the host nanotube and the hexagonal network of the CdSe chains. Such DOS data further suggest that the formation of nanocrystals inside the SWNTs cannot be explained by the consideration of the van der Waals interaction only. The calculated binding energies and DOS in this study show that the interaction between nanotubes and the CdSe crystals is non‐covalent in nature. This is further confirmed by the experimental observations (from HRTEM and Raman spectroscopy) that the tube wall was not significantly damaged. These calculations show the formation energy of CdSe nanocrystals inside the nanotube, assuming that the CdSe crystals are already available: these also show that all three crystals considered are thermodynamically stable inside the tube. In a bulk covalent material such as CdSe, changes in the bonding arising directly from low dimensionality are the dominant driving force towards structural change, and interactions with the tube wall (predominantly van der Waals) are of secondary importance. The computational calculations interpret the experimentally observed 3:3 CdSe tubes in terms of energy, compared with the other polymorphs considered. However, the magnitude of binding is dependent on the exchange correlation functional and may well change once the dispersion corrections are included; however, this aspect has not been explored in this study.

**Table 3:** Bond lengths, charges and binding energies per CdSe calculated for all three composites and isolated 1D CdSe crystals.

System	Cd−Cd [Å]	Cd−Se [Å]	Charge	*E*_binding_ [eV]
3:3@(9,9)	3.66–3.68	2.55–2.59	+0.51	−0.23
4:4@(9,9)	3.35–3.37	2.60–2.85	+0.45	−0.36
4:2@(9,9)	3.40–3.42	2.62–2.67	+0.44	−0.31
1D CdSe (3:3)	3.70–3.74	2.60–2.63	–	–
1D CdSe (4:4)	3.36–3.37	2.60–2.86	–	–
1D CdSe (4:2)	3.42–3.45	2.64–2.68	–	–

### 2.5 Biocompatibility Evaluations for Glucan‐Wrapped Nanohybrids

To evaluate the potential application of the obtained water soluble for glucan‐wrapped nanohybrids (hybrid material **3**) in biosensing devices, biocompatibility evaluations were carried out.

**Figure 14:**
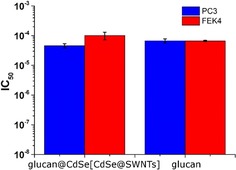
Cell viability (48 h) assays. Blue) Glucan@CdSe[CdSe@SWNTs] (hybrid material **3**) [IC_50_ = (4.58±0.76) ×10^−5^ mg mL^−1^] and β‐d‐glucan [IC_50_ = (6.72±1.08) ×10^−5^ mg mL^−1^] in PC‐3 cells under normoxic condition (21 % O_2_ and 5 % CO_2_). Red) Cell viability of glucan@CdSe[CdSe@SWNTs] (hybrid material **3**) [IC_50_ = (1.01±0.29) ×10^−4^ mg mL^−1^] and β‐d‐glucan [IC_50_ = (6.64±0.35) ×10^−5^ mg mL^−1^] in FEK4 cells under normoxic conditions. The results are reported as a mean ± standard error and represented with respect to controls.

**Figure 15:**
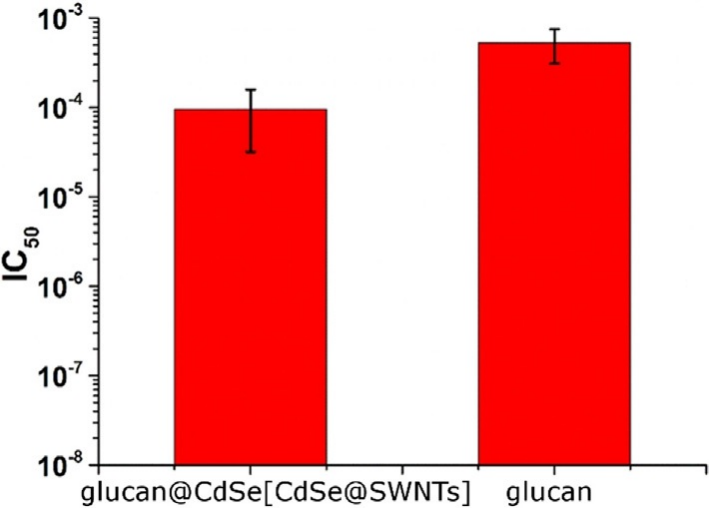
Cell viability of glucan@CdSe[CdSe@SWNTs] (hybrid material **3**) [IC_50_ = (9.54±6.36) ×10^−5^ mg mL^−1^] and β‐d‐glucan [IC_50_ = (5.30±2.00) ×10^−4^ mg mL^−1^] in PC‐3 cells under chemical induced acute hypoxia condition. The results were averaged over six measurements and reported as a mean ± standard error and represented with respect to controls (see the Supporting Information).

Fluorescent SWNTs have recently been employed to build sensor arrays capable of detecting protein efflux from microorganisms and synthetic scaffolds for biosensing devices.^15^ Therefore, in order to study the functionality, biocompatibility, and cell toxicity of our hybrid material **3**, standard cell proliferation assays such as MTT^16^, ^17^ and crystal violet^18^, ^19^, ^20^ assays were carried out by using human prostate cancer (PC‐3) and healthy human dermal fibroblasts (FEK4) cell lines. PC‐3 and FEK4 cells were incubated with the hybrid material **3** (glucan@CdSe[CdSe@SWNTs]), CdSe[CdSe@SWNTs] composites, and β‐d‐glucan for 48 h at 37 °C under normoxia (21 % O_2_ and 5 % CO_2_, Figure 14) and induced acute hypoxia conditions using standard CoCl_2_.6 H_2_O based assays^20^, ^21^, ^22^ (Figure 15).

The inhibitory concentration (IC_50_) of CdSe[CdSe@SWNTs] composites in PC3 under normoxia was evaluated by MTTs, giving an estimated IC_50_ of (9.69±1.81) ×10^−6^ mg mL^−1^ (from six measurements). For pure raw SWNTs in isolated and independent experiments, the IC_50_ was in the range of 1.17 ×10^−7^ to 6.3 ×10^−7^ mg mL^−1^ under the same conditions, showing the expected high toxicity of un‐functionalized SWNTs. This contrasts with the decreased toxicity of hybrid material **3**, which ranges from 1.08 ×10^−5^ to 7.40 ×10^−5^ mg mL^−1^, depending on cell line: HeLa or PC3. In cytotoxicity evaluations in HeLa cells, under similar conditions using covalently functionalized SWNTs, the IC_50_ was reported to be in the range of IC_50_ ≫5 ×10^−7^ m. 23

However, unexpected IC_50_ values emerged for pure CdSe crystals in independent estimations.^24^ The IC_50_ values indicated high toxicity, but we did not find them reproducible; this is likely to be due to a varying cellular uptake of samples caused by the low solubility of this inorganic material. Upon addition of the β‐d‐glucan and the formation of encapsulating strands around CdSe[CdSe@SWNTs] fibers, an increased solubility of hybrid material **3** significantly facilitated its cellular uptake. Figures 14 and 15 show the IC_50_ of CdSe@SWNTs@glucan and β‐d‐glucan required to reduce the cell growth by half. In general, a lower IC_50_ value indicates a more toxic sample. Figure 14 shows that the polysaccharide β‐d‐glucan has comparable values of IC_50_ in either FEK4 or PC‐3 cell lines, of (6.64±0.35) ×10^−5^ or (6.72±1.08) ×10^−5^ mg mL^−1^, respectively. The inhibitory concentrations of CdSe@SWNTs@glucan in PC‐3 and FEK4 differ by an order of magnitude, and their values of IC_50_ in FEK4 and PC‐3 are (1.01±0.29) ×10^−4^ and (4.58±0.76) ×10^−5^ mg mL^−1^, respectively. These results indicate that hybrid material **3** (denoted glucan@CdSe[CdSe@SWNTs]) display a higher toxicity in cancerous cells than in healthy cells, in agreement with previous studies regarding the preferential uptake of polysaccharides by cancer cells. The cytotoxicity estimations for PC‐3 cells treated with hybrid material **3** (glucan@CdSe‐ [CdSe@SWNTs]) as well as β‐d‐glucan alone, over 48 h at 37 °C under hypoxia‐induced condition, are presented in Figure 15. The viability of cancerous cells was determined by using crystal violet assays. The IC_50_ values estimated for β‐d‐glucan and (glucan@CdSe[CdSe@SWNTs]) were found to be (5.30±2.00) ×10^−4^ and (9.54±6.36) ×10^−5^ mg mL^−1^, respectively. Therefore, these estimations seem to suggest that under the conditions where PC‐3 cells were grown in conditions deprived of oxygen, mimicking the acute hypoxic conditions present within certain tumors, hybrid material **3** (glucan@CdSe[CdSe@SWNTs]) is likely to show approximately ten times more toxicity than β‐d‐glucan alone. The significantly reduced toxicity for quantum nanomaterials as well as for carbon nanotubes in the presence of this biopolymer is remarkable, and consistent with our very recent observations in new radiolabeled and simultaneously filled SWNT nanohybrids, which showed promising behavior in living systems in terms of kinetic stability and probe integrity with respect to loss of internal/external functional moieties.3c

## 3 Conclusions

For the first time, pristine SWNTs were filled with CdSe nanowires on a laboratory scale, giving rise to thus‐far elusive luminescent nanohybrids suitable for functional biomaterial investigations. Their emergence was made possible by operating at elevated temperature and in the advanced exclusion of air and moisture during the nanosynthetic protocols. The intimate structure of CdSe nanocrystals following encapsulation inside pristine SWNTs emerged by combining experimental and theoretical investigations. One batch of SWNTs was as made by electric arc growth (Arc‐SWNT) and the other by chemical vapor deposition (CVD‐SWNT). The filling yield, estimated by HRTEM is high (above 60 %), and is comparable with that normally expected for such solid‐state encapsulation techniques. The new obtained CdSe@SWNTs hybrids were characterized by X‐ray powder diffraction, high‐resolution transmission electron microscopy, and Raman spectroscopy. X‐ray diffraction together with HRTEM and EDX analyses confirm the filling of the SWNTs with a hexagonal CdSe phase. The lower interplanar crystal phase of the CdSe filling the SWNT, in comparison with the spacing for a free hexagonal CdSe, indicates that CdSe inside the CNTs is confined and the structure is compressed in order to adapt to the internal space of the CNT. We have also found a high‐frequency shift of the stretching modes in Raman. This shift of the Raman bands might imply charge transfer from SWNTs to CdSe, in which CdSe nanocrystals are the acceptor. DFT calculations validated the predictions on possible structures for CdSe that can be found inside the SWNT under the experimental conditions applied. The geometries of three different 1D CdSe nanowire stands were optimized from different starting structures. The lowest energy structure was found to be the 3:3 form, whereas the next thermodynamically stable (with lowest minimum energy) structure was 4:2. The calculated binding energies show that, although all three polymorphs may be found experimentally, the most thermodynamically stable structure (3:3) is directly comparable to the CdSe structures widely observed experimentally inside these carbon nanotubes. DFT calculations in the gas phase indicate that there is no significant modification or damage found in the SWNT walls under the influence of CdSe, which is consistent with experimental observations from Raman spectroscopy and X‐ray diffraction. This seems to suggest that interactions with the tube wall, predominantly van der Waals interactions, are of secondary importance in stabilizing the resulting geometry under confinement. The reasons for the formation of nanowires in a CNT template environment are likely to be kinetic, as well as thermodynamic, in nature. The binding energy and charge transfer between CdSe crystals and the SWNTs were modeled by using DFT, and this information may assist experimentalists in their further structural interpretations of semiconductors nanoparticles inside SWNTs. Finally, the inhibitory concentration (IC_50_) of a new luminescent, functional material denoted glucan@CdSe[CdSe@SWNTs] (hybrid material **3**) in healthy and cancerous cells was evaluated. These studies showed that, under normoxia and hypoxia conditions, hybrid material **3** shows only marginally increased toxicity for cancer cells with respect to the pristine polysaccharide fibers of β‐d‐glucan, highlighting the fact that the use of such adjuvants can mediate the high cellular toxicity of toxic materials such as CdSe@SWNTs and also allows the complete characterization of the encapsulated materials in water media.

This work sheds light on the nature of encapsulated nanocrystals in hierarchical and biocompatible CdSe—SWNT–glucan hybrids by using techniques situated at the interface between materials chemistry research and life sciences, such as confocal fluorescence imaging and biocompatibility assays. Structural predictions of the luminescent nanowires formed under confined conditions in the solid state are validated by DFT calculations combined with structural materials investigations techniques in bulk, on thin films, and on the nanoscale.

## Experimental Section

Arc‐ and CVD‐synthesized SWNTs were available from Carbolex (with average diameters of 1–2 nm) or from Thomas Swann (with average diameters of 1.4–5 nm). Steam refinement of raw Arc‐ and CVD‐synthesized SWNTs was used here as the method of choice to minimize the presence of external defects and functional groups into the scaffold whilst simultaneously opening the tubes.

### Advanced Purification and CdSe Filling of SWNTs

The purification of as‐made CVD SWNTs (Thomas Swann, Elicarb) was achieved by introducing steam carried by an argon flow through the reactor. SWNTs (500 mg) were introduced in a quartz reactor (9 mm diameter) placed inside of the quartz tube (5 cm diameter) and within the alumina lining of a furnace tube, which was flushed with argon for at least 1 h prior to the experiment to minimize the presence of air. Argon‐degassed steam was introduced by bubbling argon through boiling water. The furnace temperature was gradually increased to 900 °C over approximately 30 min and the reaction was maintained for at least 20 min and no longer than 2 h, depending on the sample size. The resulting sample was refluxed in 16 % HCl overnight, which removes most of the metal catalyst, and washed with NaOH and double‐distilled water on a 2 μm membrane filter until the filtrate was neutral pH. A further toluene washing/filtration step was carried out in order to obtain the highest purity SWNTs after filtration. Prior to filling, the tubes were standardized and dispersed by sonication at room temperature for 10 min. We found that, when arc‐tubes were used, only a very small purification yield was obtained. Together with CdSe, the nanotubes were then kept in a Schlenk tube and heated at 50 °C above the melting point of CdSe (CdSe, Sigma–Aldrich, mp. 1268 °C) for 12 h. The annealing process was carried out under an inert atmosphere by using standard Schlenk techniques and glassware in order to exclude air and moisture from the reaction vessel. The resulting CdSe[CdSe@SWNTs] materials were refluxed in HCl for 1 h, washed with a 0.1 m EDTA/H_2_O solution, and finally heated at 180 °C, giving a purified CdSe[CdSe@SWNTs] hybrid material **1**. To obtain hybrid material **2 (**CdSe@SWNTs), **1** was refluxed in 0.1 m aqueous HCl for 1 h, washed with Milli‐Q water, and heated at 180 °C. Hybrid material **3** was obtained by dispersing CdSe[CdSe@SWNTs] (**1**) in water (1 mg mL^−1^). β‐d‐Glucan from barley (1 mg mL^−1^) was heated and sonicated (15 min) in DMSO. The two suspensions were mixed, sonicated for 15 min, and stirred for 2 h. The resulting composites were dried under reduced pressure for 12 h and re‐dispersed in distilled water (2 mL). The SWNT‐containing samples (ca. 20 % by weight) were isolated from H_2_O after approximately 3 h of centrifugation. The aqueous layer was removed by a freeze‐drying procedure and the samples denoted (glucan@CdSe[CdSe@SWNTs]) were re‐dispersed in EtOH for TEM/HRTEM or AFM analysis.

### Powder X‐ray Diffraction

Analyses of the crystalline structures and phase identification were performed by X‐ray diffraction (XRD Bruker D8 ADVANCE) with a monochromatized source of Cu K_α_1 radiation (*λ*=1.5406 nm) at 1.6 kW (40 KV, 40 mA). The samples were prepared by placing a drop of a concentrated ethanol dispersion of particles onto a single‐crystal silicon plate.

### Transmission Electron Microscopy (TEM)

TEM images were obtained with a Gatan Dual‐vision digital camera on a JEOL 1200EXII transmission electron microscope coupled with EDX spectroscopy (point resolution, 0.16 nm). The operating voltage was 120 kV. HRTEM images were obtained from the sample (deposited on a Lacey carbon film‐copper grid, purchased from Agar Scientific) on a transmission electron microscope (JEM‐2100 LaB6 or JEOL 2100 FEGTEM) operated at 100 kV. The differing imaging approaches and repeated experiments showed a very high batch‐to‐batch consistency of the nanohybrids synthesized. For the glucan‐coated nanocomposite (hybrid material **3**) and free CdSe, as well as for the uncoated hybrid materials **1** and **2**, HRTEM images were also recorded on a JEOL 3000F field‐emission gun instrument. This instrument is equipped with an Oxford Instruments EDX spectrometer with a super atmospheric thin window (SATW) detector that allows chemical analysis of elements down to boron under suitable conditions. In addition, a Gatan imaging filter (GIF) equipped with a 2k 794IF/20 MegaScan CCD camera allows chemical analysis using electron energy loss spectroscopy (EELS).

Generally, for glucan‐wrapped SWNTs (filled or unfilled), all examined samples showed an excess of organic materials forming a film, so TEM imaging of the glucan‐wrapped CdSe filled SWNTs was rather challenging. Nevertheless, AFM imaging confirmed the presence of the glucan‐wrapped material **(**hybrid material **3**).

### Raman Spectroscopy

Raman spectroscopy was carried out on a Renishaw inVia Raman spectroscope. The measured samples used in this project normally contained carbon nanotubes, graphene oxide, or reduced graphene oxide. The specimens were either in the solid state or dispersed in pure water (Milli‐Q) or a water/ethanol (1:1) mixture. During the measurement, carbon nanomaterials samples were deposited on an aluminum plate substrate. In some cases, silicon wafers or a glass lens were also used as the substrate. The input wavelength was set at 830 nm for SWNTs and their composite samples. For the hybrid **1** nanocomposite and its glucan derivative, spectra at 514 nm were also recorded. More than ten repeats were applied in the Raman spectroscopy measurements to achieve sufficient signal‐to‐noise ratios, and the beam was focused on at least three different positions across the specimen; these spectra were averaged to obtain representative peaks of the sample.

### Atomic Force Microscopy (AFM)

AFM images were recorded on a Digital Instruments Multimode SPM instrument with a Nanoscope IIIa controller. This was operated in tapping mode with a “J” scanner having a lateral range of approximately 100 μm and a vertical range of 6 μm. Silicon probes (Nascatec GmbH model NST NCHFR) were used, with resonant frequencies of approximately 320 kHz. Calibration of the AFM was accomplished by scanning a 10 μm pitch with 200 nm 3D reference from Digital instruments. Contact‐mode AFM was found to be unsuitable (despite its better lateral resolution with respect to tapping‐mode AFM), as it physically removed any deposits from the area scanned.

### Solid‐State NMR

The solid‐state ^13^C NMR spectrum of pristine steam‐purified SWNTs was recorded at the Inorganic Chemistry Laboratory, University of Oxford, using a mixture of SWNTs spiked onto a bulk, anhydrous KBR sample that was homogenized by grinding in a glove box under N_2_. Spectra were recorded on a BRUKER AVANCE III HD 400 spectrometer equipped with a 4 mm magic angle spinning (MAS) probe at 101 MHz at room temperature (298 K). All NMR spectra were acquired by using MAS with a spinning speed of 10 kHz and were fully interpreted for this type of SWNTs for the first time.

### Confocal Microscopy

Confocal fluorescence microscopy was performed by using a Nikon eclipse T*i*‐E inverted microscope with 60× oil objective lens, equipped with an LU‐N laser unit and three continuous visible lasers (405.0, 488.0, 561.0 nm). All images were processed by using functions within the NIS elements software package. Thin films of relevant samples were prepared by drying out their suspensions onto a horizontal surface.

### Computational Details

First principles DFT, as implemented in the CASTEP code,^11^ was employed to optimize structures and plot densities of states of SWNTs containing different 1D CdSe polymorphs. The exchange and correlation interactions are described by using generalized gradient approximation (GGA) parametrized by Perdew, Burke, and Ernzerhof (PBE).^14^ Ultrasoft pseudopotentials were generated by using the “on‐the‐fly” formalism in CASTEP. A plane‐wave basis set with the energy cutoff of 320 eV was used to expand the wave function. Structure optimizations were performed using the BFGS algorithm and the forces on the atoms were obtained from the Hellman–Feynman theorem including Pulay corrections. In all optimized structures, forces on the atoms were smaller than 0.05 eV Å^−1^. A single *k*‐point (Γ) was used for all calculations on molecular CdSe crystals and for the CdSe@SWNT composites. In calculations on CdSe with periodic boundary conditions, reciprocal space was sampled at between 1–5 *k*‐points by using the method of Monkhorst and Pack.^25^

## Conflict of interest

*The authors declare no conflict of interest*.

## Supporting information

As a service to our authors and readers, this journal provides supporting information supplied by the authors. Such materials are peer reviewed and may be re‐organized for online delivery, but are not copy‐edited or typeset. Technical support issues arising from supporting information (other than missing files) should be addressed to the authors.

Supplementary
