# Supplementary material for: Encapsulation of Cadmium Selenide Nanocrystals in Biocompatible Nanotubes: DFT Calculations, X‐ray Diffraction Investigations, and Confocal Fluorescence Imaging
Source: ChemistryOpen. 2018 Jan 18;7(2):144–58. doi: 10.1002/open.201700184 (PMC5792830; doi:10.1002/open.201700184)
Supplement: Supplementary file 1 — Supplementary [file OPEN-7-144-s001.pdf]

ChemistryOPEN

Supporting Information

© 2018 The Authors. Published by Wiley-VCH Verlag GmbH & Co. KGaA, Weinheim

Encapsulation of Cadmium Selenide Nanocrystals in Biocompatible Nanotubes: DFT Calculations, X-ray Diffraction Investigations, and Confocal Fluorescence Imaging

David G. Calatayud+,[a, b] Haobo Ge+,[a] Navaratnarajah Kuganathan+,[c] Vincenzo Mirabello+,[a] Robert M. J. Jacobs,[d] Nicholas H. Rees,[d] Craig T. Stoppiello,[e] Andrei N. Khlobystov,[e] Rex M. Tyrrell,[f] Enrico Da Como,[g] and Sofia I. Pascu*[a]

open_201700184_sm_miscellaneous_information.pdf

Table of content

1. Single-photon emission spectroscopy and laser-scanning confocal fluorescence microscopy

2. Confocal microscopy

2. Energy-dispersive X-ray spectroscopy (EDX) of SWNTs.

3. Computational Details.

4. Scanning electron microscope (SEM)

5. Transmission electron microscopy

6. Raman spectroscopy

7. Computational details

8. Diffusion-Ordered NMR Spectroscopy

9. Solid-state ^{13}C NMR of pristine SWNTs

10. MTT and crystal violet assays

11. UV-vis-NIR spectroscopy

12. References

1. Single-photon emission spectroscopy and laser-scanning confocal fluorescence microscopy

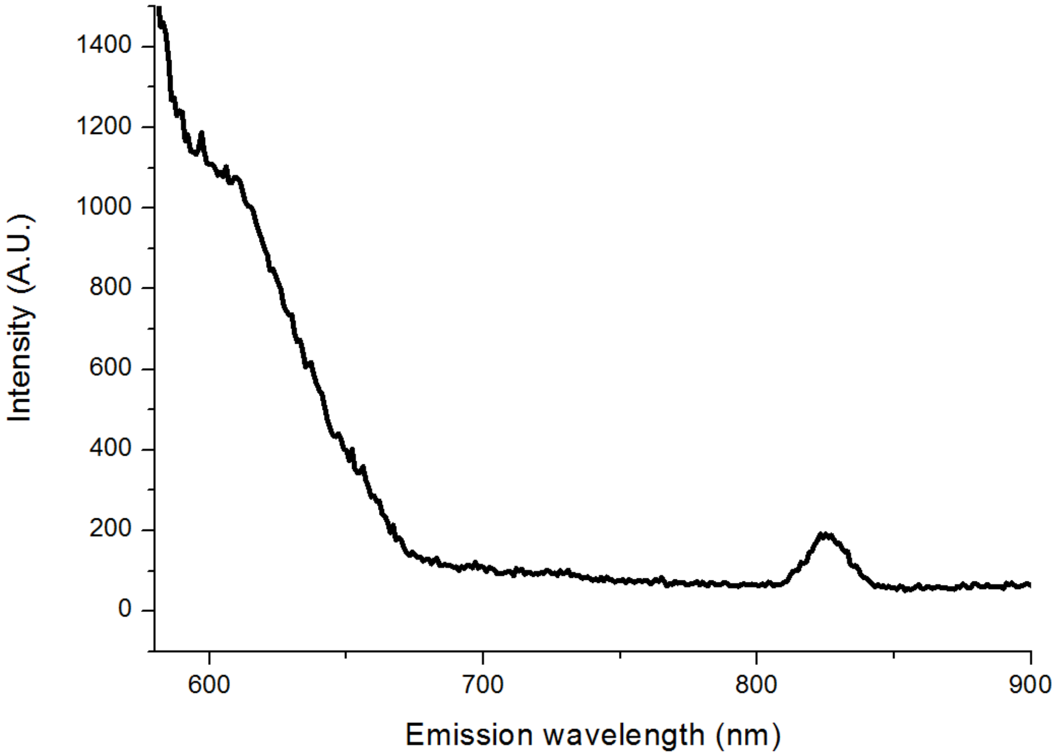

Figure S1. Fluorescence emission spectrum: at λex 550 nm: CdSe@SWNT hybrid 1 (1 mg composite) dispersed in glucan (20mg-3ml in DMSO:H2O) showing a nearIR emission with λmax 825 nm.

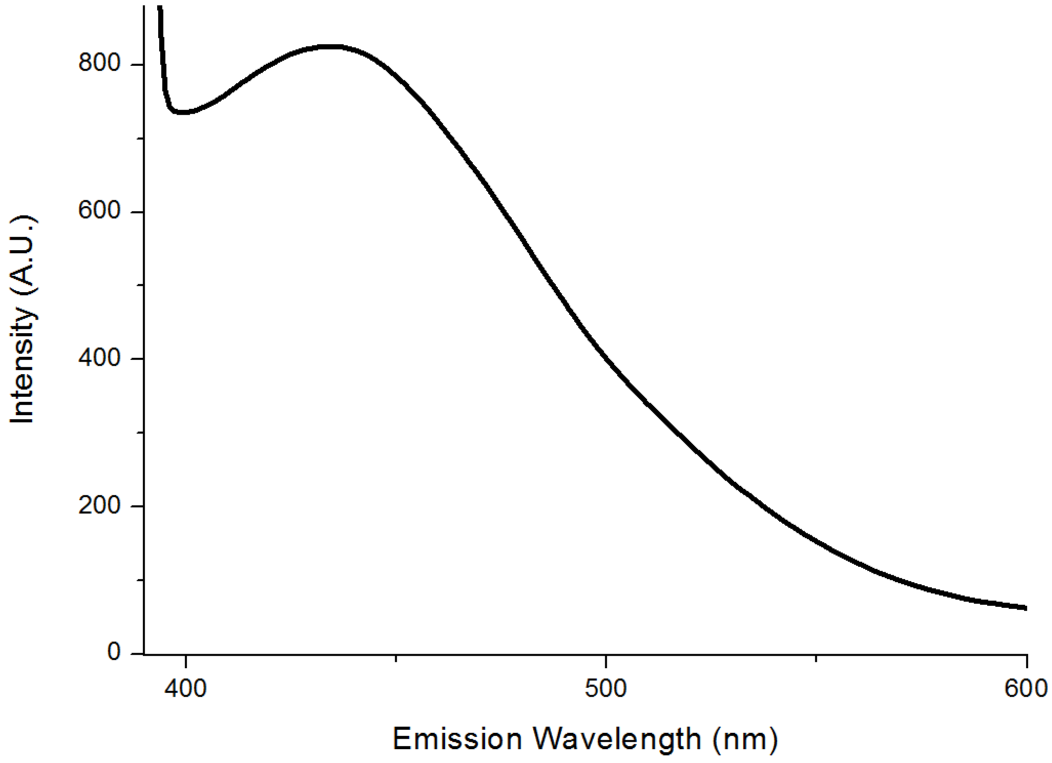

Figure S2. Fluorescence emission spectrum at λ_{ex} = 380 nm: CdSe@SWNT hybrid 1 (1 mg composite) dispersed in the presence of beta-D-glucan (20 mg in 3ml in DMSO:H_2O).

2. Confocal microscopy.

Confocal fluorescence microscopy: Imaging was performed using a Nikon eclipse T*i*-E inverted microscope system with 60X oil objective lens, equipped with LU-N laser units and three continuous visible lasers (405.0, 488.0 561.0 nm). All images were processed using functions within the NIS elements software package. Thin films of relevant samples were prepared by drying out their suspensions onto the horizontal surface of glass bottom microwell dishes (35mm petri dish 10mm microwell MatTek).

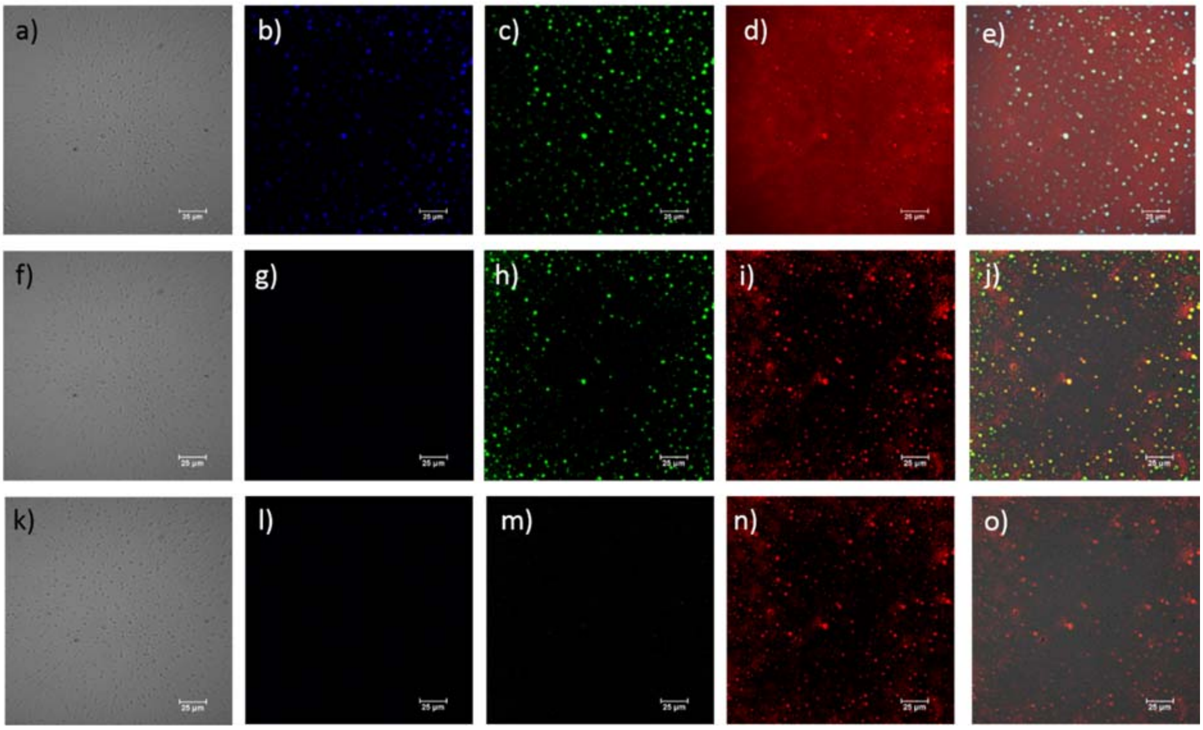

Figure S3. Single-photon laser-scanning confocal microscopy of CdSe thin film of a chloroform suspension (0.1 mmol in 3 mL of CHCl3) drying out on a horizontal surface. a-e) λ_{ex} = 405.0 nm; f-j) λex = 488.0 nm; k-o) λ_{ex} = 561.0 nm. a, f, k) DIC channel; b, g, l) blue channel (λem = 417-477 nm); c, h, m) green channel (λ_{em} = 500-550 nm). D, i, n) red channel (λ_{em} = 570-750 nm). e, j, o) overlay of the blue-green-red channels. Scalebar: 25μm.

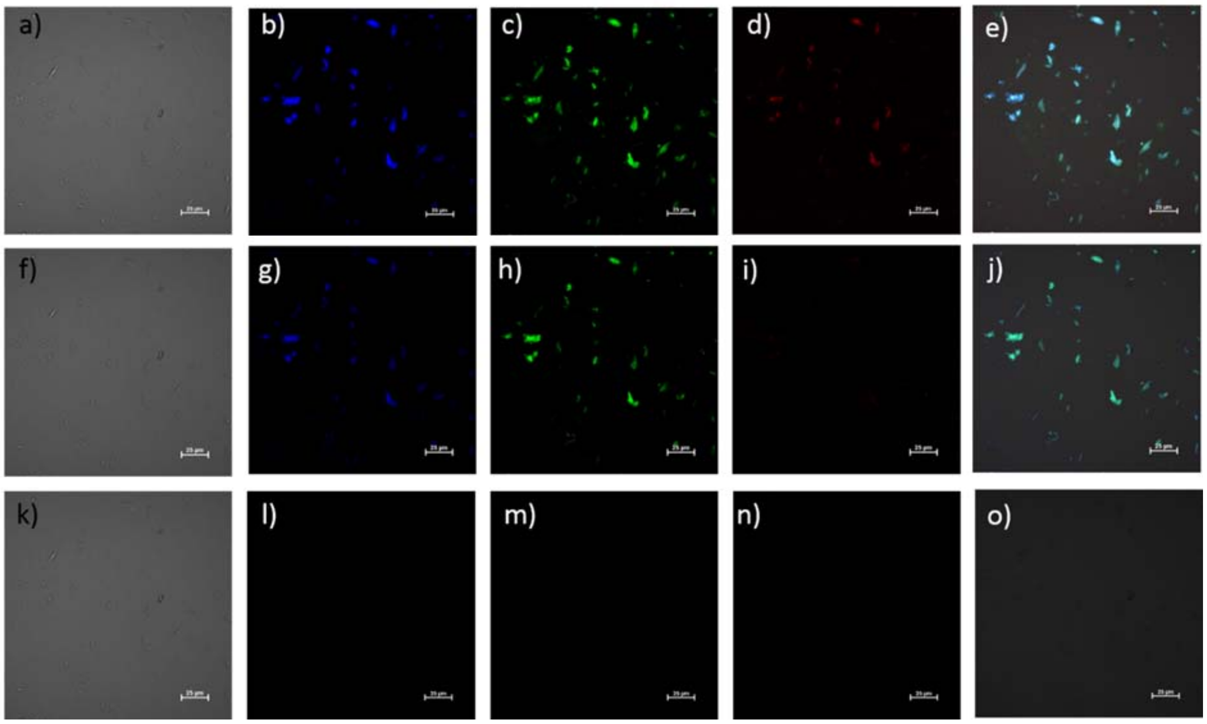

Figure S4. Single-photon laser-scanning confocal microscopy of Lumidots™ CdSe/ZnS 480 nm thin film of a toluene suspension drying out on a horizontal surface. a-e) λ_{ex} = 405.0 nm; f-j) λ_{ex} = 488.0 nm; k-o) λ_{ex} = 561.0 nm. a, f, k) DIC channel; b, g, l) blue channel (λ_{em} = 417-477 nm); c, h, m) green channel (λ_{em} = 500-550 nm). D, i, n) red channel (λem = 570-750 nm). e, j, o) overlay of the blue-green-red channels. Scalebar: 25µm.

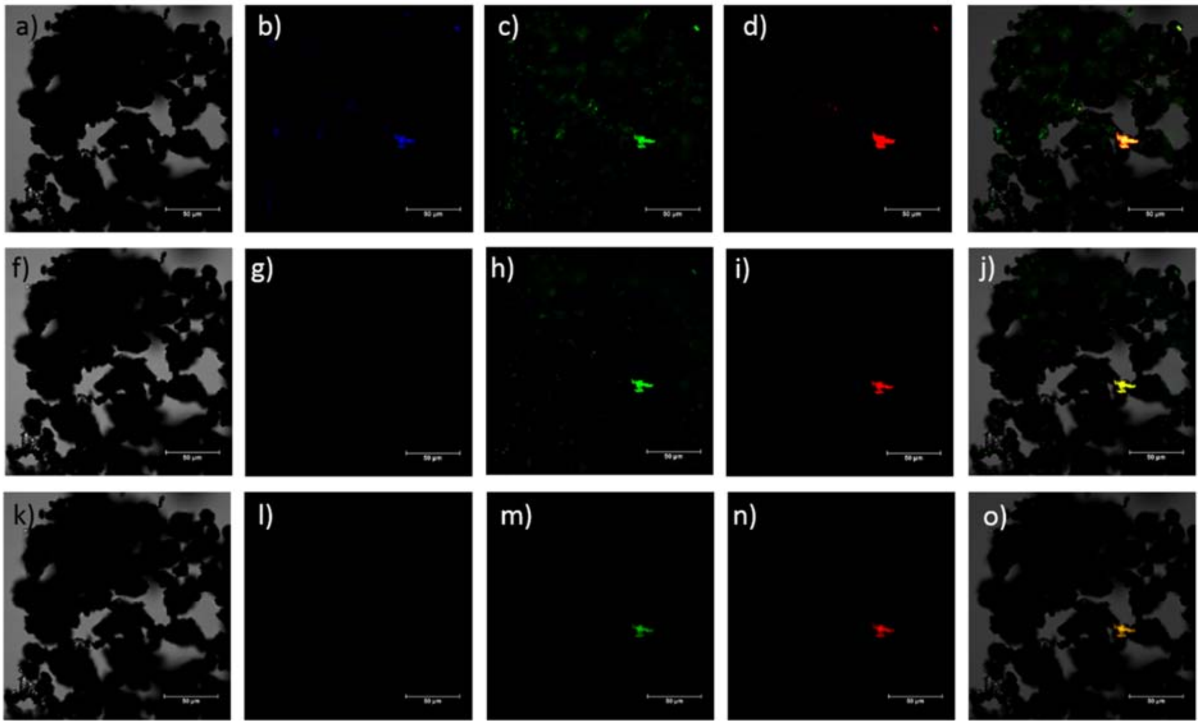

Figure S5. Single-photon laser-scanning confocal microscopy of CdSe@SWNTs. a-e) λ_{ex} = 405.0 nm; f-j) λ_{ex} = 488.0 nm; k-o) λex = 561.0 nm. a, f, k) DIC channel; b, g, l) blue channel (λ_{em} = 417-477 nm); c, h, m) green channel (λ_{em} = 500-550 nm). D, i, n) red channel (λ_{em} = 570-750 nm). e, j, o) overlay of the blue-green-red channels. Scalebar: 50µm

3. Energy-dispersive X-ray spectroscopy (EDX) of SWNTs.

Figure S6. EDX of CVD-SWNTs sample post-purification, showing traces of Fe, presumably remaining (from the catalysis used in CVD production of the Elicarb SWNts) after steam purification.

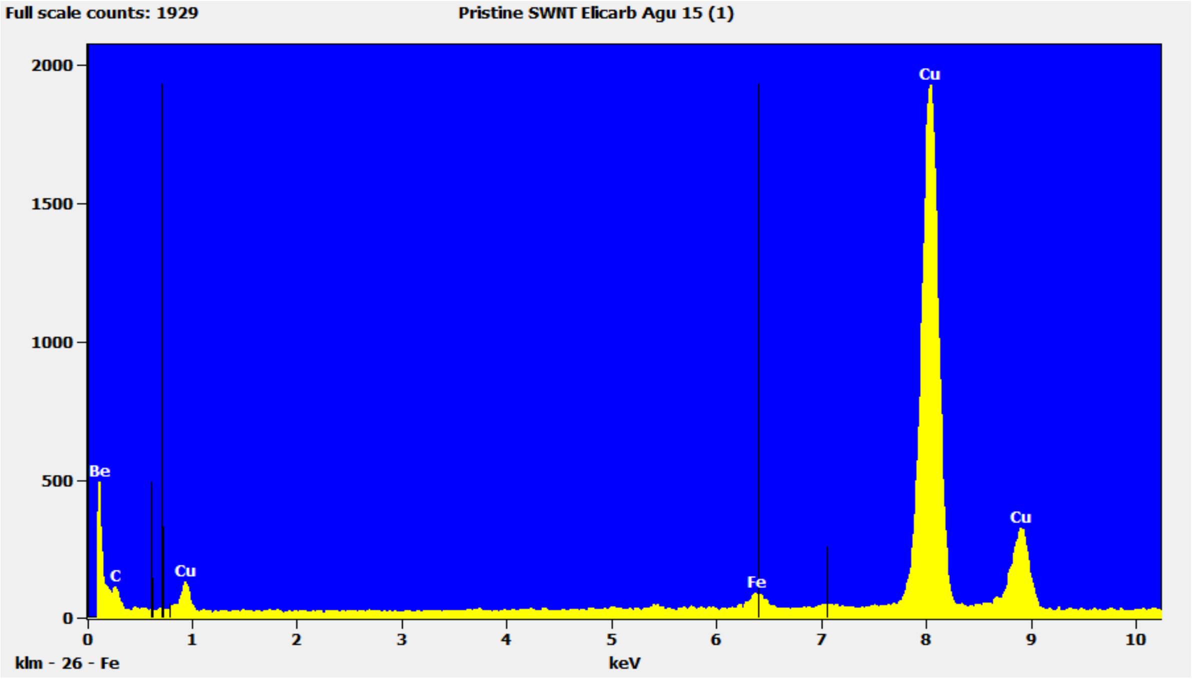

Table S1. EDX-corresponding quantitative results of CVD-SWNTs sample post-purification

**Element line**	**Net Counts**	**Weight %**	**Weight % Error**	**Atom %**	**Atom % Error**
Be K	1578	70.9	± 5.4	79.0	± 6.1
C K	1065	24.1	± 2.6	20.2	± 2.2
Fe K	934	0.1	± 0.0	0.0	± 0.0
Cu K	38069	4.9	± 0.1	0.8	± 0.0
Total		100.0		100.0	

4. Scanning electron microscope (SEM)

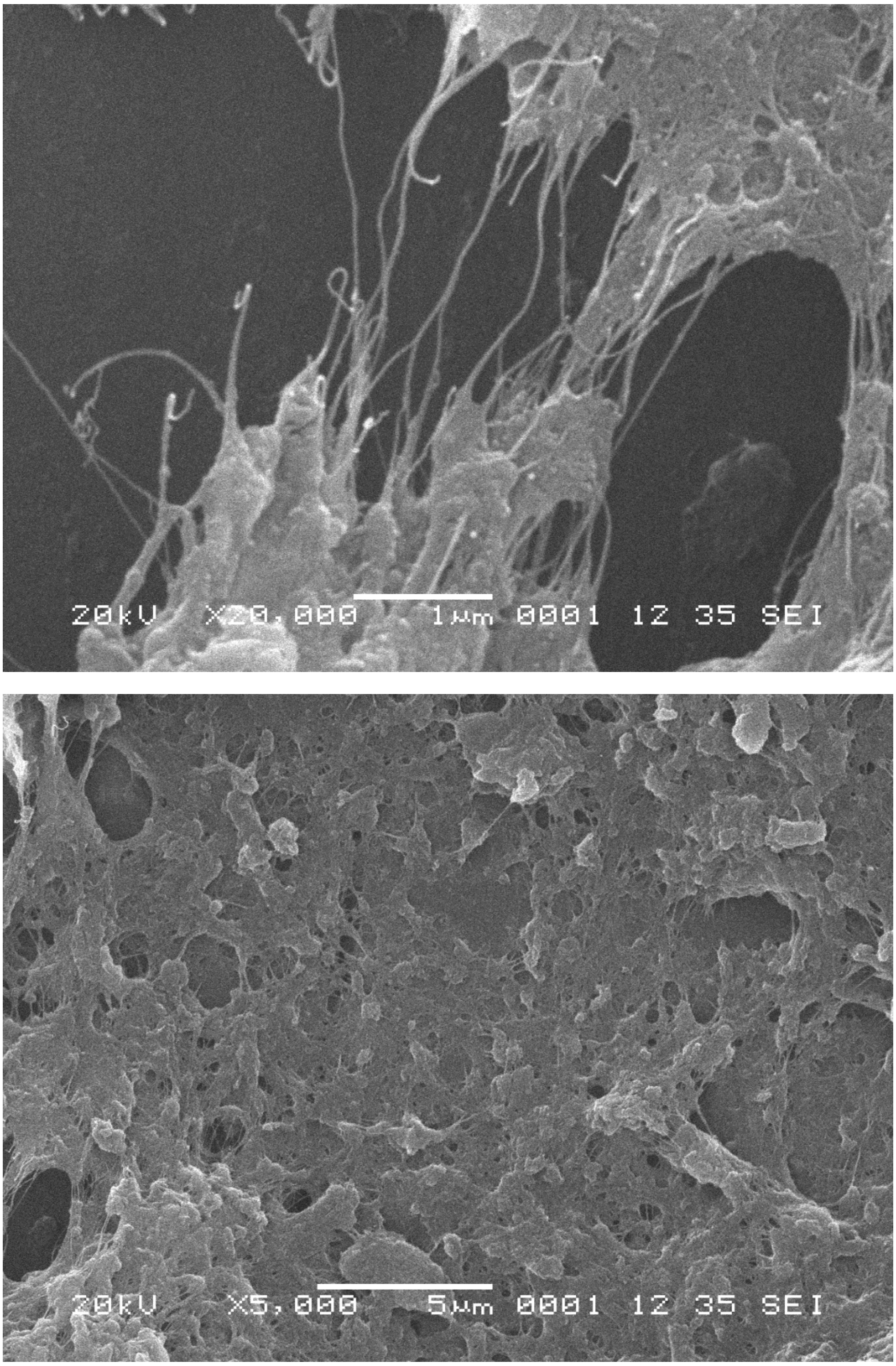

Figure S7. Typical SEM of the glucan-dispersed CVD-SWNTs strands and general morphology of the composite, at different magnifications.

5. Transmission electron microscopy

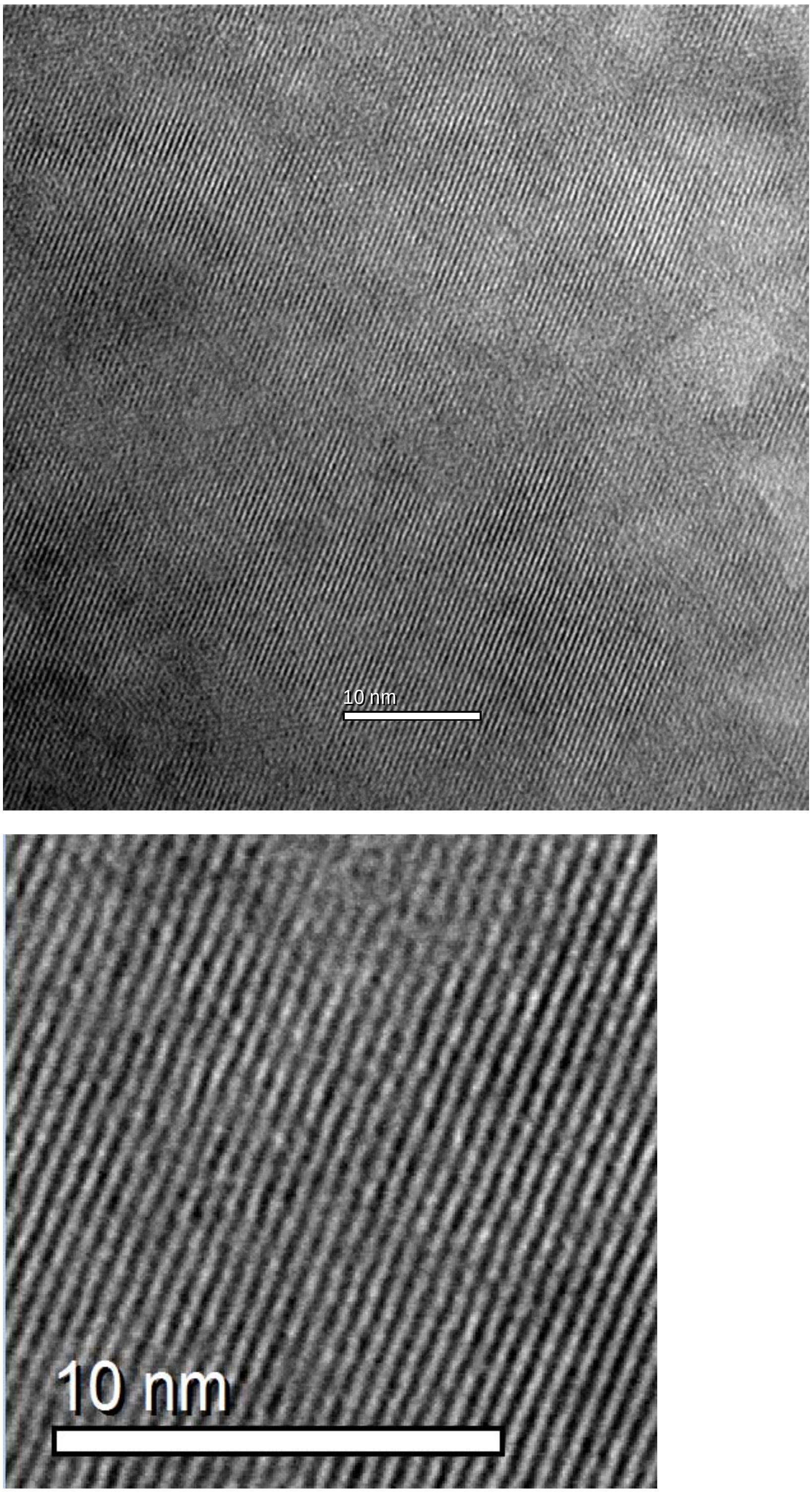

Figure S8. HRTEM of free CdSe annealed under reduced pressure at 1320 ºC for 12 h, in the absence of SWNTs, as a control experiment.

Conditions: 10 mg of CdSe powder with mp 1268˚C was mixed in a glove box with arc-synthesised and acid-purified SWNTs (1 mg) in a 10:1 (weight ratio). The grinding of the mixture was carried out in a glove box with the total exclusion of air and moisture, and it was placed in a quarts tube fitted with a Young's tap manifold. This was placed in a Schlenk line, evacuated under reduced pressure and sealed. The tube was placed in a tree-zone Carbolite furnace an heated up at ca. 50˚C above MPt over 12hrs, then allowed to cool down to the room temperature. The content of the quarz tube was recoveded, washed three times with dil. HCl and water in a sequence of 3 filtrations/redispersion and 3 sonication cycles no longer than 5 min. Then, TEM sample was prepared by redispersion at 1mg/ml conc in EtOH and analyzed on a lacey copper grid.

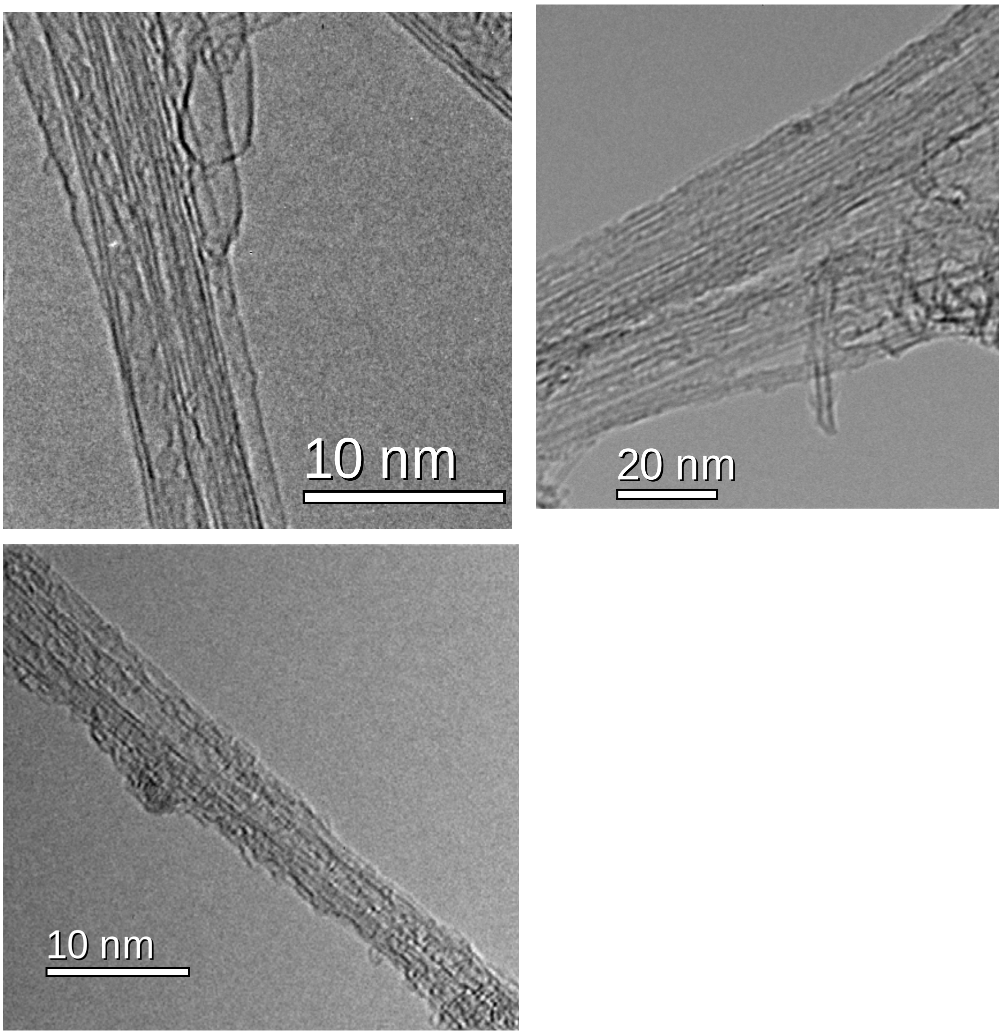

Figure S11. Alternative HRTEM images for arc-made SWNTs filled with CdSe.

Figure S12. Alternative TEM images for CVD-made SWNTs filled with CdSe after advanced purification by EDTA washing recorded at different magnifications.

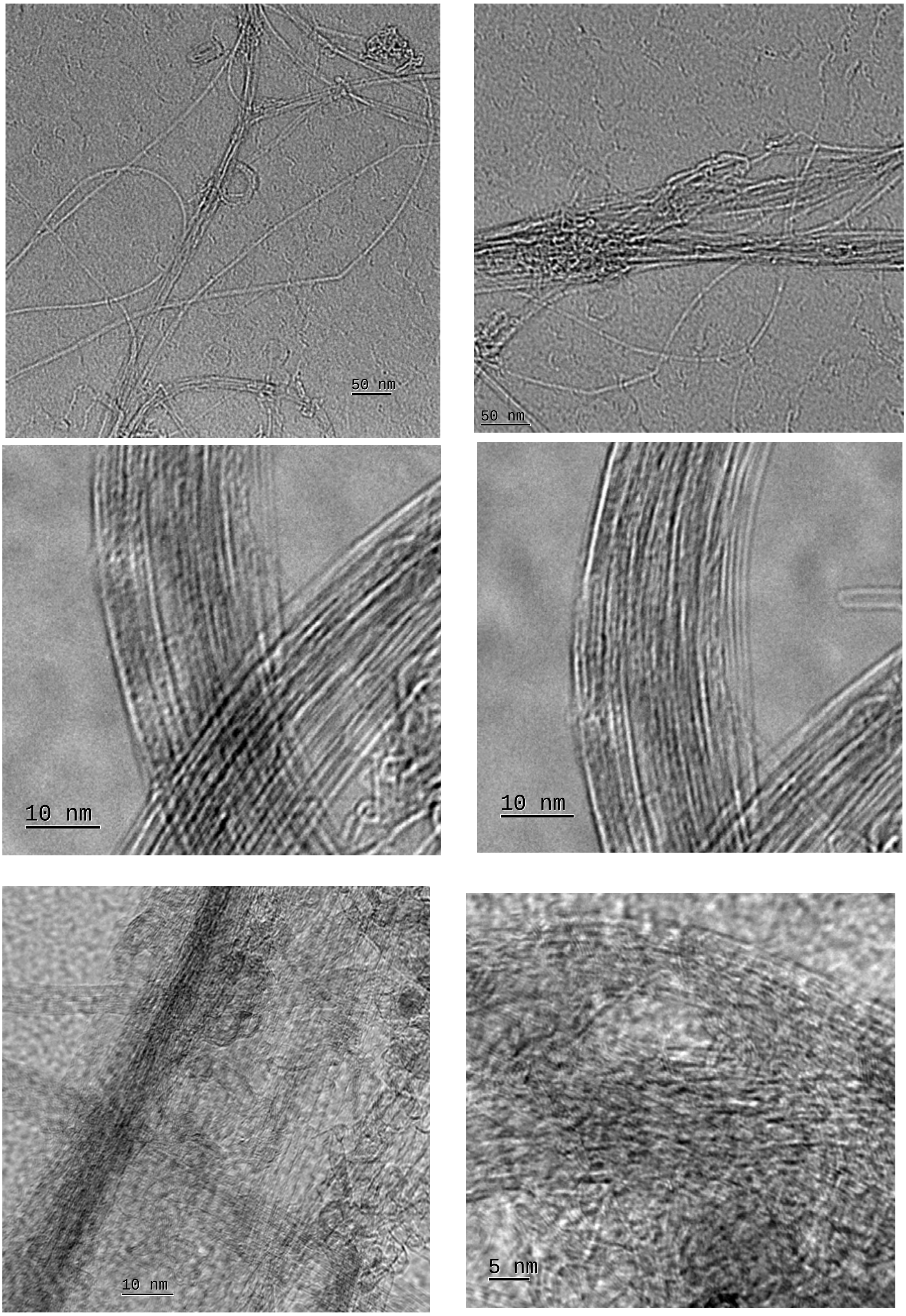

Figure S13. Alternative TEM and HRTEM images for CVD-made SWNTs filled with CdSe after advanced purification by EDTA washing, recorded at different magnifications.

Some 1% DWNTs were observed in Thomas Swann Elicarb ultrapure tubes, alongside mainly (10,10) diameter SWNTs even after advanced purification by steam methods.

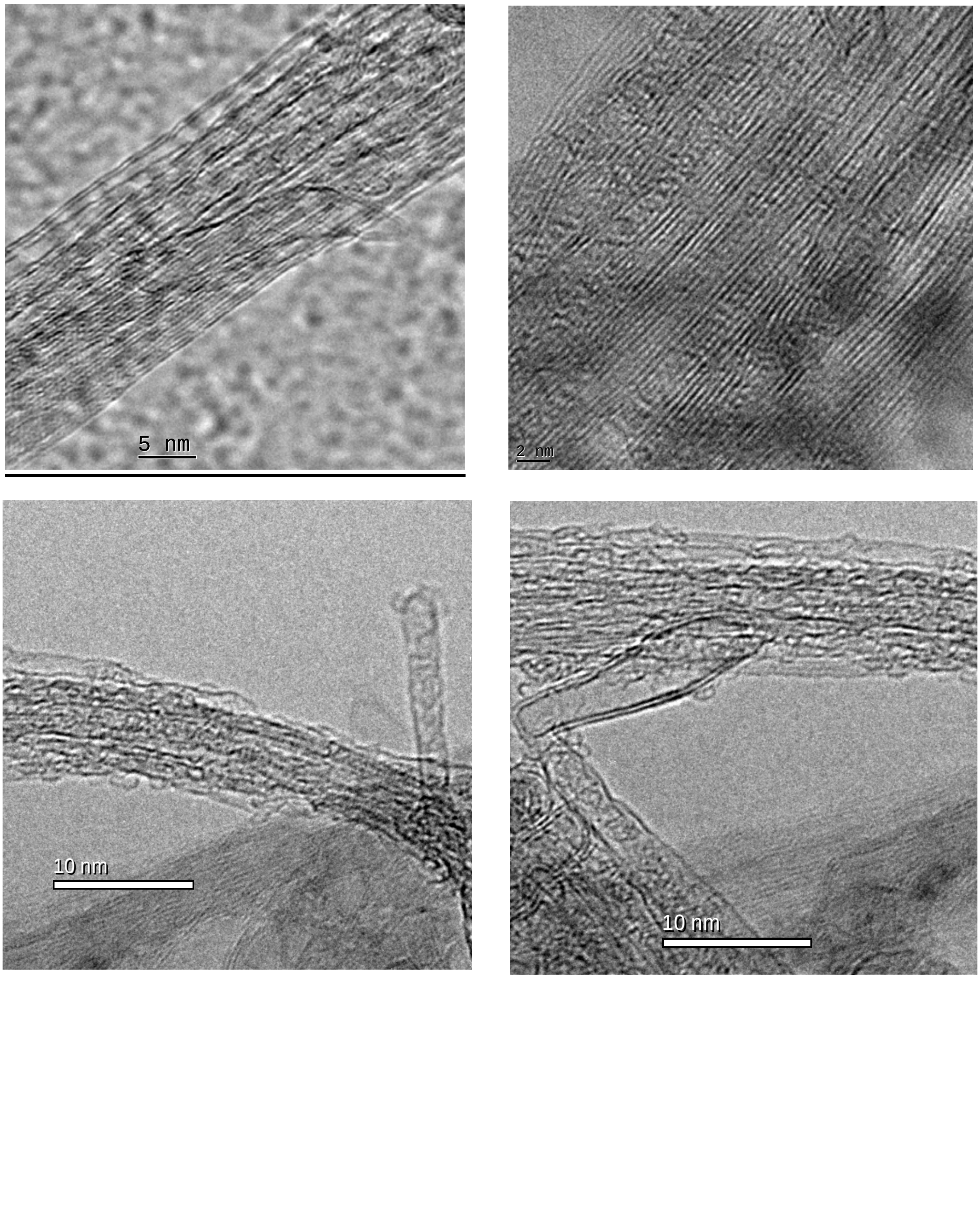

Figure S14. EDX of CdSe filled CVD-SWNTs sample Hybrid 1, recorded on a copper/nickel grid.

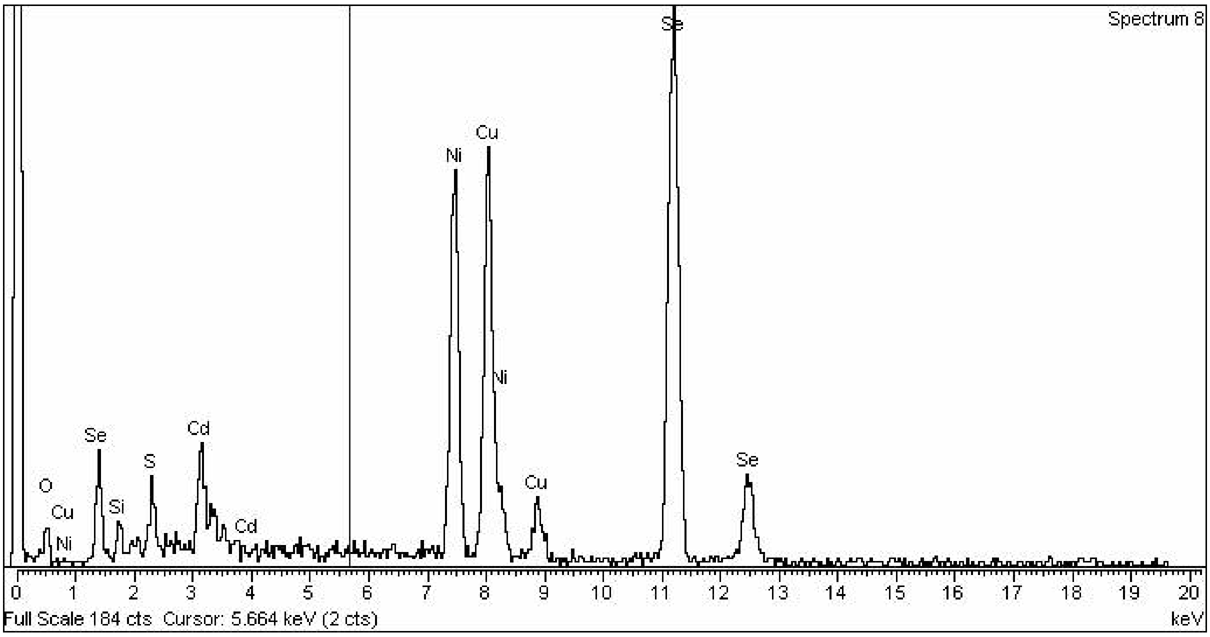

Figure S15. Alternative HRTEM images for CVD-made SWNTs filled with CdSe after advanced purification by EDTA washing, recorded at different magnifications.

Note that some 5% MWNTs were observed in Thomas Swann Elicarb ultrapure tubes, alongside mainly (10,10) diameter SWNTs even after advanced purification by steam methods.

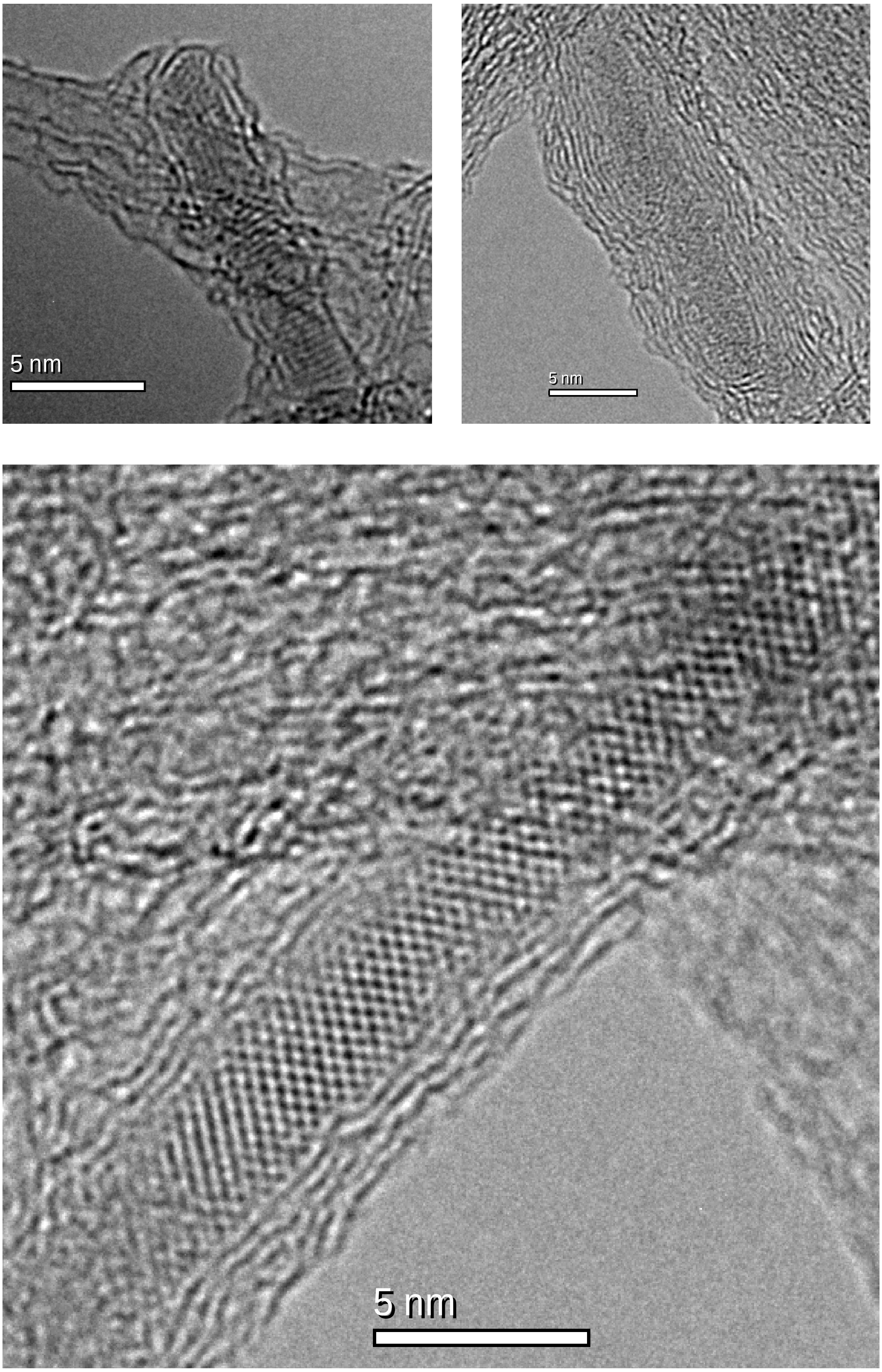

6. Raman spectroscopy

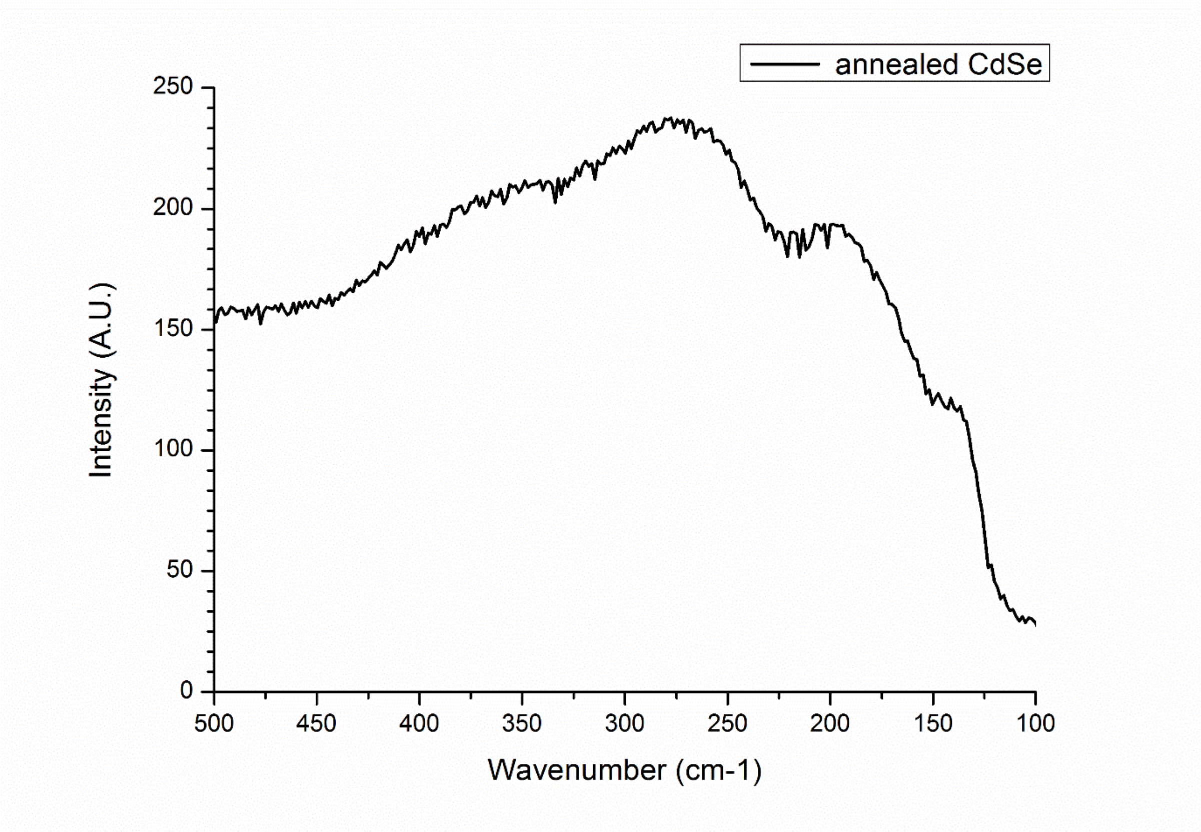

Figure S9. Raman spectroscopy (830 nm) of free CdSe annealed under reduced pressure at 1320 ºC for 12 h, in the absence of SWNTs, as control experiment

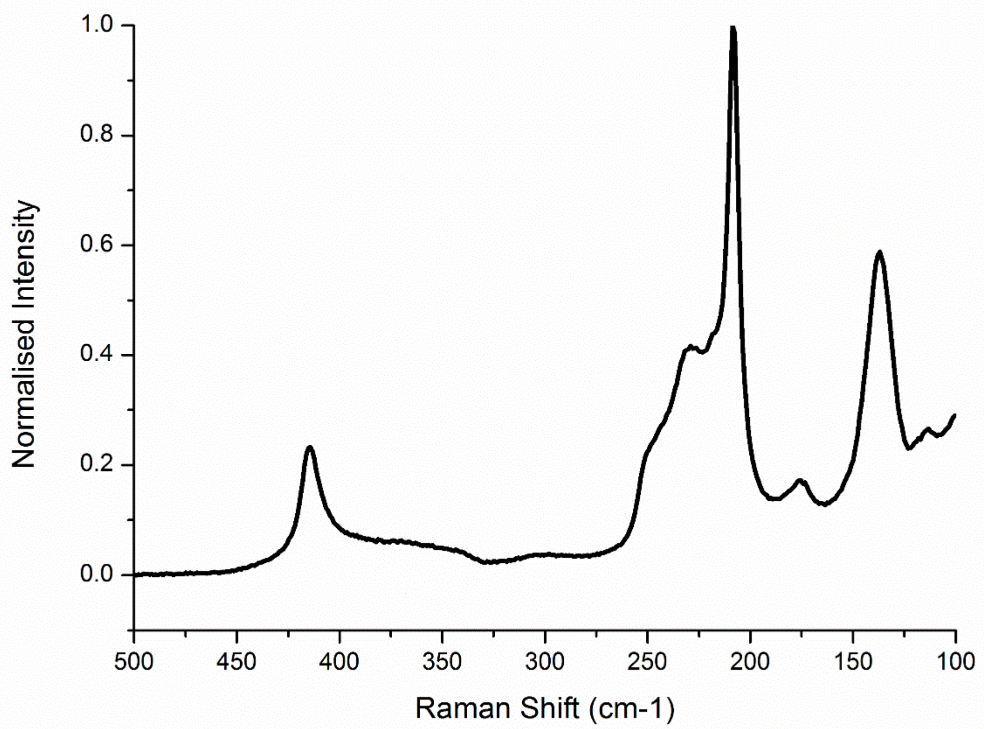

Figure S10. Raman spectroscopy (830 nm) of bulk, free CdSe (Aldrich).

7. Computational Details

<u>Figure S16: DFT – Optimised geometries of representative nano structures</u>

A comparison Experimental - DFT calculated:

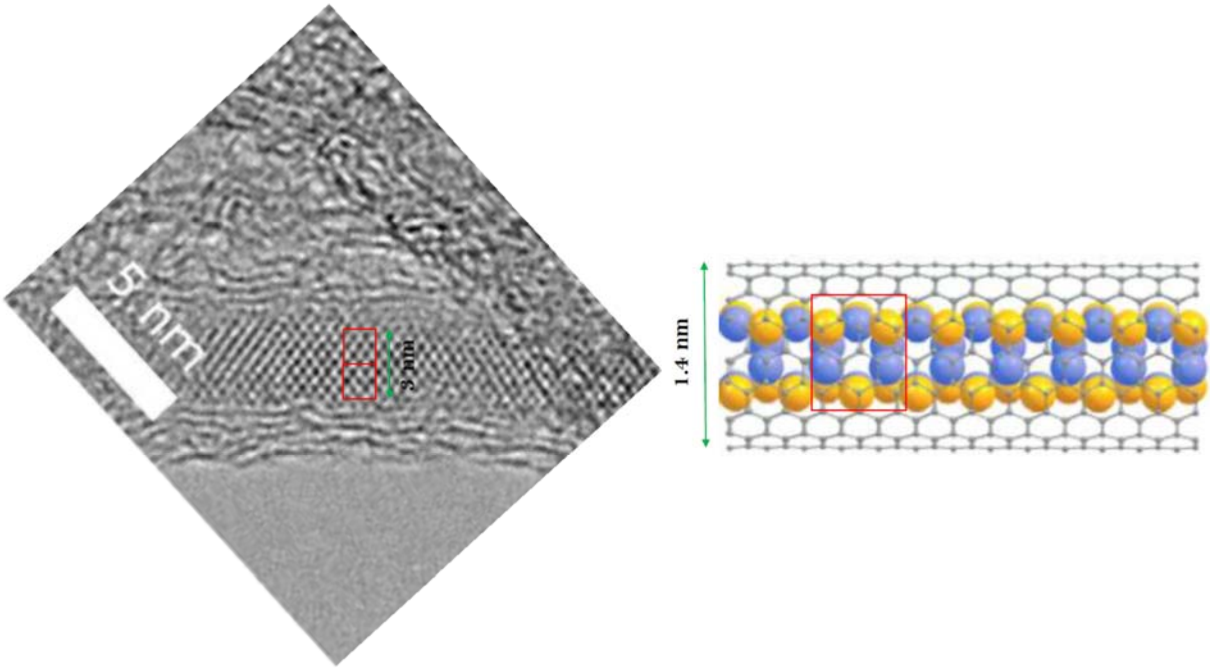

A. CdSe (33)@(8,8)

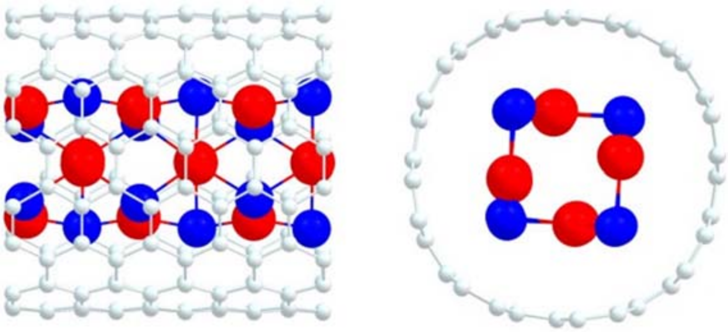

B. CdSe (33)@(9,9)

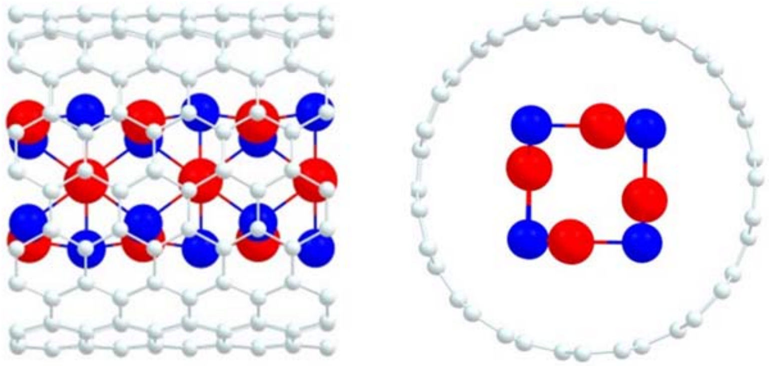

C. CdSe (33)@(10,10)

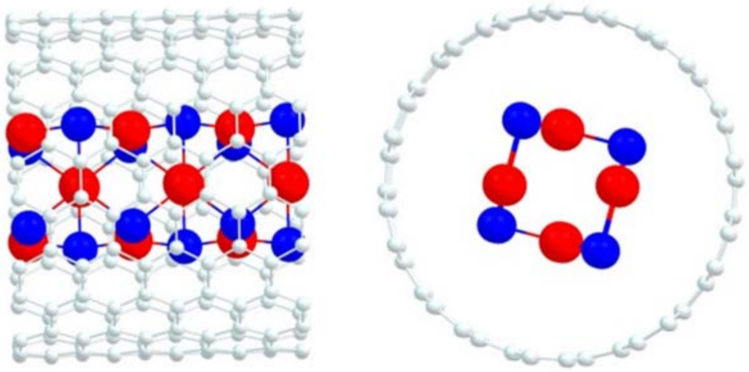

D. CdSe (44)@(8,8)

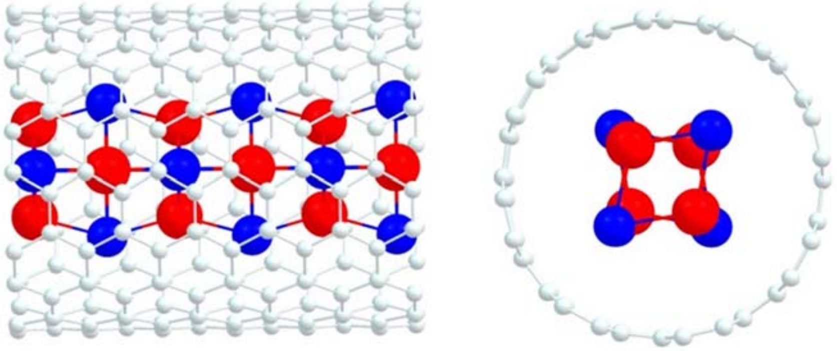

E. CdSe (44)@(9,9)

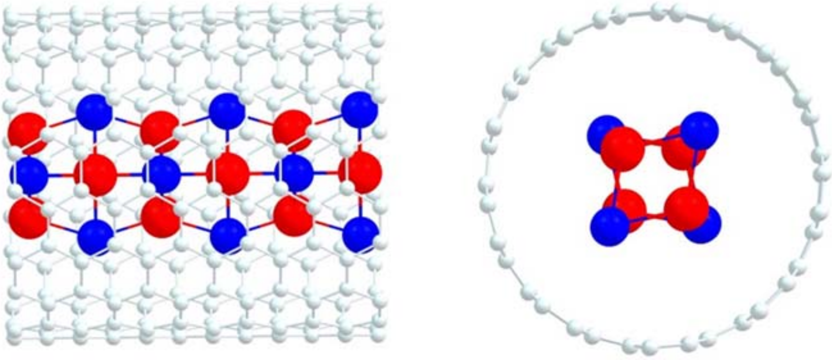

F. CdSe (44)@(10,10)

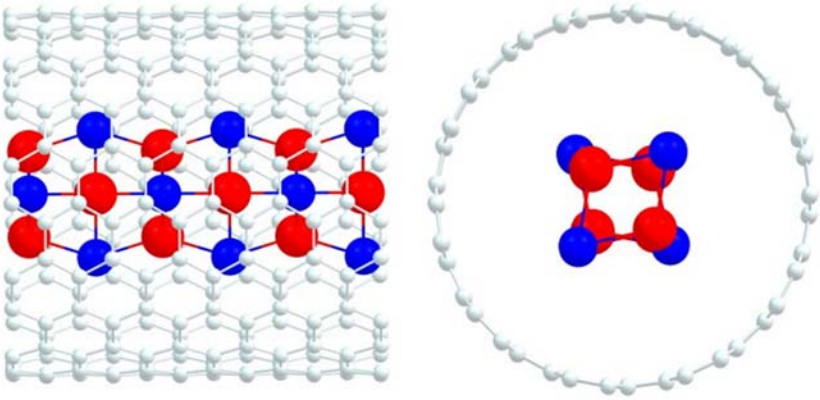

G. CdSe (42)@(8,8)

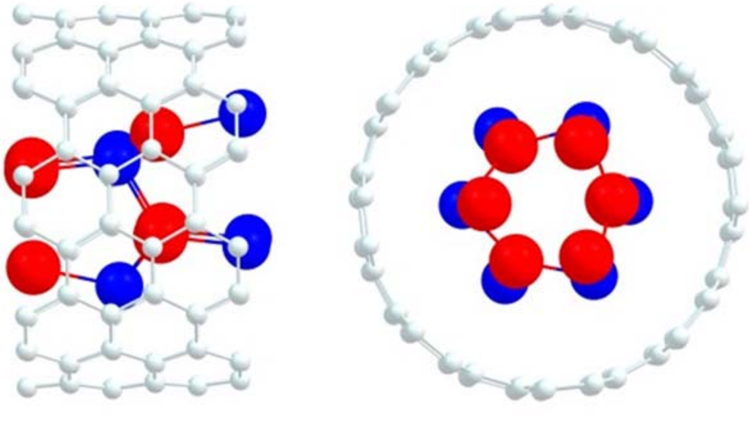

H. CdSe (42)@(9,9)

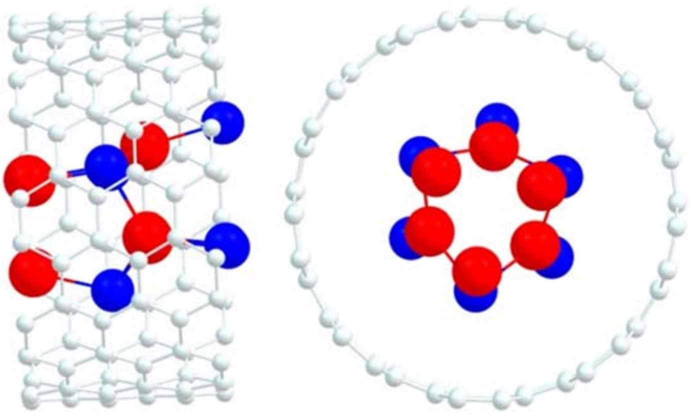

I. CdSe (42)@(10,10)

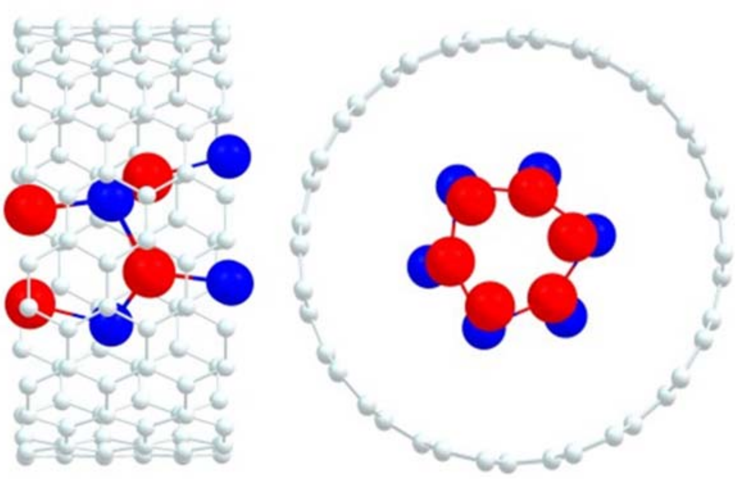

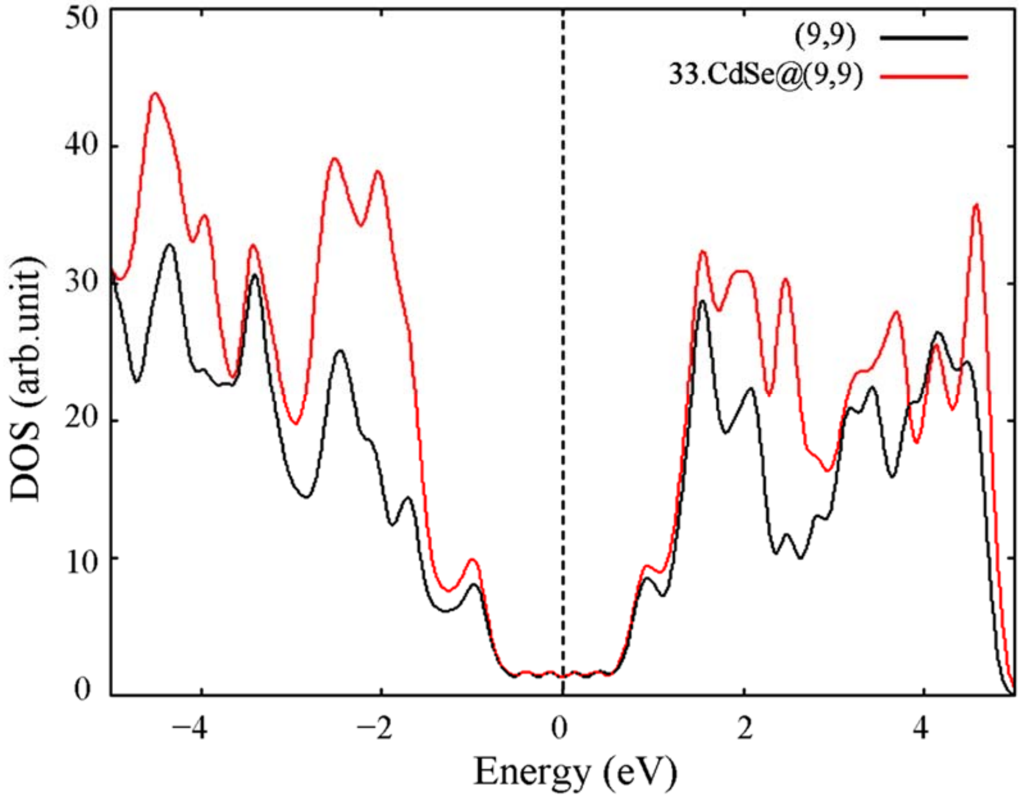

Figure S17. Density of states plots for 33.CdSe@(9,9). The black lines correspond to the pristine (9,9) tube and the red lines to the tube plus interaction of the 33.CdSe crystal. The zero corresponds to the Fermi energy.

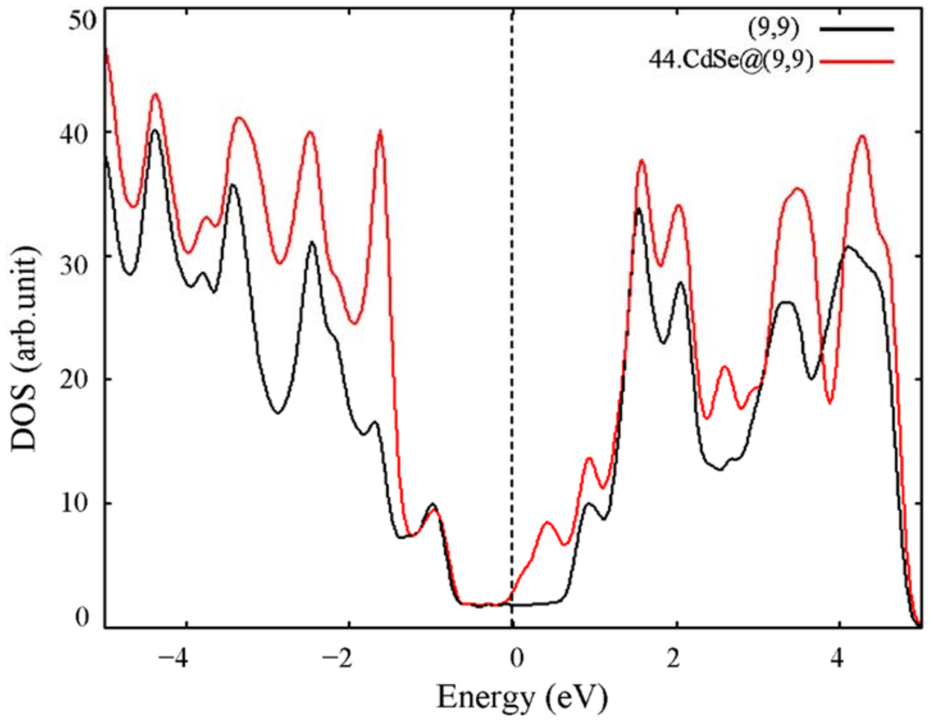

Figure S18. Density of states plots for 44.CdSe@(9,9). The black lines correspond to the pristine (9,9) tube and the red lines to the tube plus interaction of the 44.CdSe crystal. The zero corresponds to the Fermi energy.

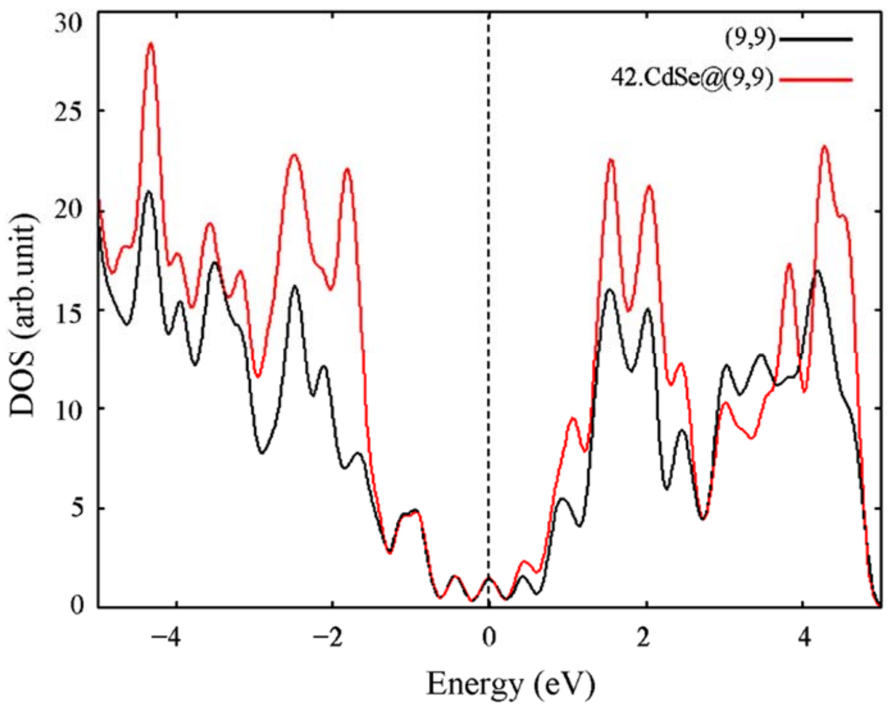

Figure S19. Density of states plots for 42.CdSe@(9,9). The black lines correspond to the pristine (9,9) tube and the red lines to the tube plus interaction of the 42.CdSe crystal. The zero corresponds to the Fermi energy.

Table S2. Composition of supercells used to model CdSe@SWNT

System	Diameter of nanotubes (d_t) (Å)	Number of carbon atoms in the pristine nanotube	Number of atoms in the CdSe crystal	Number of atoms in the CdSe@SWNT
33@(8,8)	10.85	160	24	184
33@(9,9)	12.36	180	24	204
33@(10,10)	13.56	200	24	224
44@(8,8)	10.85	192	24	216
44@(9,9)	12.36	216	24	240
44@(10,10)	13.56	240	24	264
42@(8,8)	10.85	96	12	108
42@(9,9)	12.36	108	12	120
42@(10,10)	13.56	120	12	132

Table S3. Bond lengths, charges and binding energies per CdSe calculated for CdSe CdSe (3:3)@SWNT and for an isolated 1-d 3:3 CdSe crystal

System	Cd-Cd/Å	Cd-Se/Å	Charge	$E_{binding}$/eV
44@(8,8)	3.35 −3.36	2.58 −2.79	+0.51	−0.21
44@(9,9)	3.35 −3.37	2.60 −2.85	+0.45	−0.36
44@(10,10)	3.36 −3.37	2.60 −2.86	+0.35	−0.32
1-d CdSe (4:4)	3.36 −3.37	2.60 −2.86	−	−

Table S4. Bond lengths, charges and binding energies per CdSe calculated for CdSe CdSe (4:4)@SWNT and for an isolated 1-d 4:4 CdSe crystal

System	Cd-Cd/Å	Cd-Se/Å	Charge	Ebinding/eV
42@(8,8)	3.32 −3.36	2.55−2.59	+0.49	+0.04
42@(9,9)	3.40 −3.42	2.62 −2.67	+0.44	−0.31
42@(10,10)	3.43 −3.45	2.64 −2.68	+0.35	−0.35
1-d CdSe (4:2)	3.42 −3.45	2.64 −2.68	−	−

Table S5. Bond lengths, charges and binding energies per CdSe calculated for CdSe CdSe (4:2)@SWNT and for an isolated 1-d 4:2 CdSe crystal

System	Cd-Cd/Å	Cd-Se/Å	Charge	Ebinding/eV
42@(8,8)	3.32 −3.36	2.55−2.59	+0.49	+0.04
42@(9,9)	3.40 −3.42	2.62 −2.67	+0.44	−0.31
42@(10,10)	3.43 −3.45	2.64 −2.68	+0.35	−0.35

8. Diffusion-Ordered NMR Spectroscopy

DOSY NMR spectra were recorded on a Bruker Avance 500MHz spectrometer. ^{1}H chemical shifts are referenced to tetramethylsilane. DOSY experiments were performed by using ledbpgp2 pulse sequence outlined within the Bruker library. DOSY FIDs were processed by using Topspin3.5 and then fitted on BRUKER Dynmic Center software.

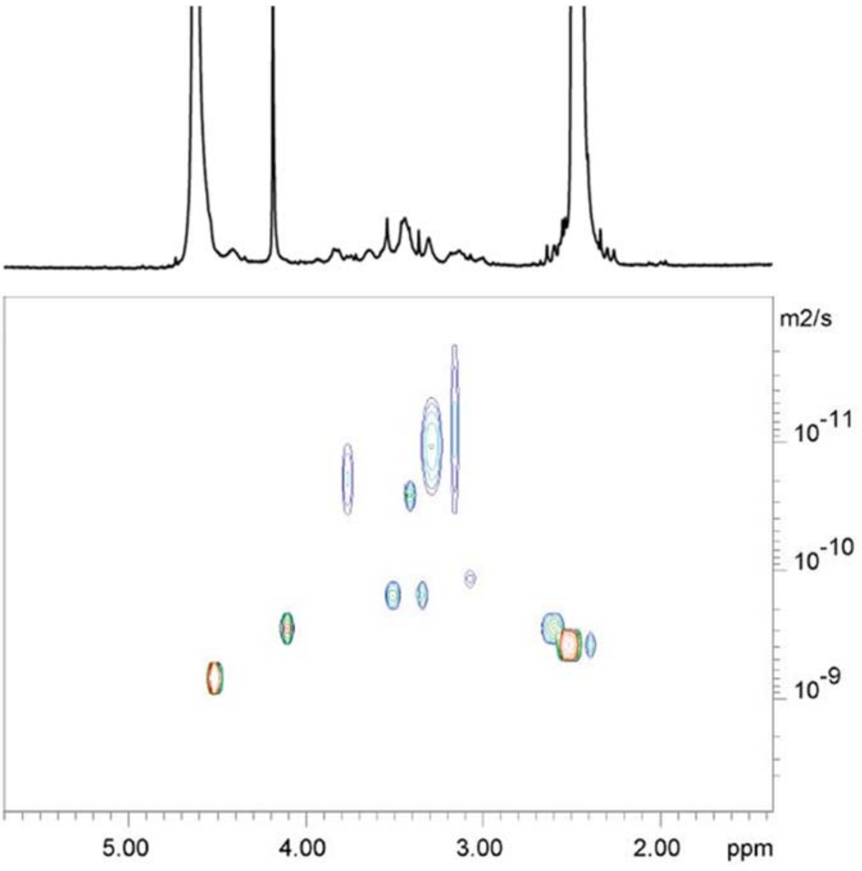

Figure S20. ^{1}H DOSY NMR spectrum (500 MHz, 298 K, D_2O) of β-D-glucan@CdSe[CdSe@SWNT]. Diffusion coefficients (D_c): δ = 4.51 ppm, D_c = 7.29 x 10^{-10} ± 1.58x10^{-13} m^2 cm^{-1} (D_2O residual of the solvent); δ = 4.10 ppm, D_c = 7.29 x 10^{-10} ± 1.58x10^{-12} m^2 cm^{-1}; δ = 3.76 – 2.54 ppm, D_{c1} = 1.93 x 10^{-11} ± 1.25x10^{-11} m^2 cm^{-1}, D_{c2} = 3.01 x 10^{-10} ± 8.00x10^{-12} m^2 cm^{-1}, D_{c3} = 1.61 x 10^{-10} ± 1.06x10^{-11} m^2 cm^{-1} , D_{c4} = 2.66 x 10^{-11} ± 3.80 x 10^{-12} m^2 cm^{-1} , D_{c5} = 1.80 x 10^{-10} ± 1.22 x 10^{-11} m^2 cm^{-}, D_{c6} = 1.22 x 10^{-11} ± 7.77 x 10^{-12} m^2 cm^{-1}, D_{c6} = 7.90 x 10^{-12} ± 1.04 x 10^{-11} m^2 cm^{-1}, D_{c7} = 1.23 x 10^{-10} ± 1.54 x 10^{-11} m^2 cm^{-1}, D_{c8} = 3.12 x 10^{-10} ± 1.12x10^{-11} m^2 cm^{-1}; δ = 2.50 ppm, D_c = 4.06 x 10^{-10} ± 1.12x10^{-11} m^2 cm^{-1}.

9 Solid-state ^{13}C NMR of pristine SWNTs

Solid-state ^{13}C NMR spectrum was recorded on a BRUKER AVANCE 400 spectrometer equipped with a 4 mm MAS probe at 101 MHz and room temperature (298 K). All spectra were acquired using magic angle spinning (MAS) with spinning speeds of 10 kHz.

Relevant Structural properties such as carbon nanotubes can be extrapolated from solid state ^{13}C NMR investigations. Diameter and number of concentric walls of CNTs directly influence the chemical shift of the carbon NMR resonances. The solid-state ^{13}C NMR spectrum was fitted by using MestReNova v10 0.2-15465 (Figure S21). A convolution operation was applied resulting in a fitted spectrum (red) in which three distinct Gaussian peaks (blue) overlap with each other. Chemical shifts, intensity and associated areas under the curve for the three resonances are reported in Table S6 for comparison.

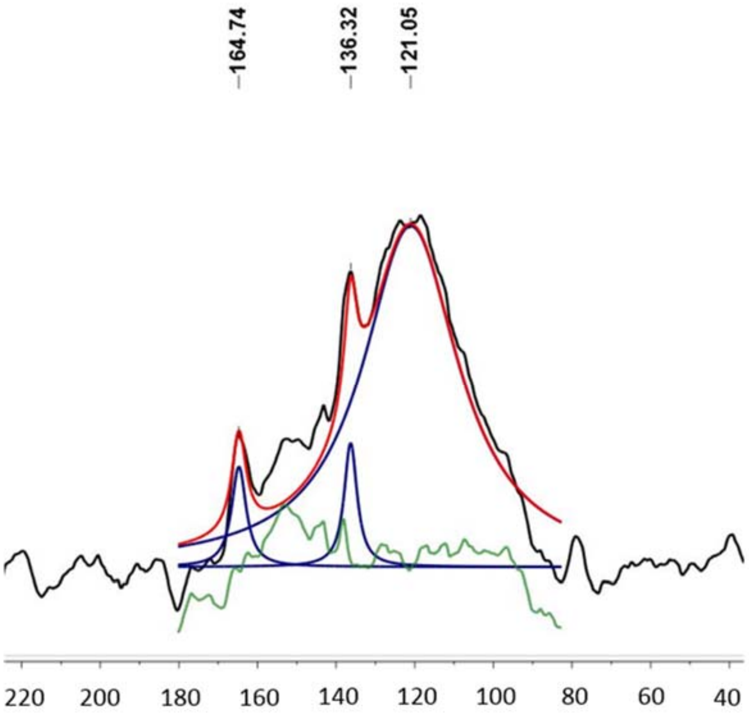

Figure S21. Solid state^{13}C NMR (298 K, 10k) spectrum of pristine SWNTs (black line), fitted ^{13}C NMR spectrum (red line), convolution peaks (blue lines and fitting residues (green line).

Table S6.

	Intensity (a.u.)	Area	Normalised (%) Area
**121.0**	24762	79280880.38	91.5
**136.3**	9009	386423.06	4.5
**164.7**	24762	352472.65	4

The peak centred at 121 ppm represent the 91.5% of the whole sample. Its chemical shift is consistent with those shifts, ranging between 118.8 and 123.8 ppm, reported for single-walled carbon nanotubes (SWNTs).[1] The average diameter of our SWNTs was also extrapolated from the following equation:

$$\delta = \frac{18.3}{D} + 102.5 = \text{ppm relative to TMS}$$

where δ is the ^{13}C NMR isotropic chemical shift. The average diameter of the SWNTs was, therefore, found to be 0.99 ± 0.20 nm. The resonances at 136.3 and 164.7 ppm may suggest the presence of impurities or structural defect or presence of C-O bounds that overall affect the 9.8% of the whole sample.

10. MTT and crystal violet assays

MTT: Cells were cultured at 37 °C in a humidified atmosphere in the air and diluted once confluence had been reached. Culture occurred in Eagle's Minimum Essential Medium (EMEM) containing 15 % foetal calf serum (FCS), 0.5 % penicillin/streptomycin and 1 % L-Glutamine. The surplus supernatant containing dead cell matter and excess protein was aspirated. The live adherent cells were then washed with 2×10 mL aliquots of phosphate buffer saline (PBS) solution to remove any remaining media containing FCS, which inactivates trypsin. Cells were re-suspended in solution by incubation in 3 mL of trypsin–PBS solution (0.25 % trypsin) for 5 min at 37 °C. After trypsinisation, 5 mL of medium containing serum was added to inactivate the trypsin and the solution was centrifuged for 5 min (1000 rpm, 25 °C) to remove any remaining dead cell matter. The supernatant liquid was aspirated and 5 mL of medium was added to the cell matter left behind. Cells were counted using a haemocytometer and then seeded as 0.15 million cells in absence of indicator dyes such as phenol red in cell medium (15 % FCS), for 48 h in poly-D-lysine coated dishes.[2,3]

Chemical induced hypoxia: 290 mg of $CoCl_2 \cdot 6H_2O$ was dissolved into 50 mL of milliq water to form a 25 mM stock solution. Then 40 µL of the stock solution was added into 9.96 mL of 10% RPMI serum medium to achieve 100 µM concentration. Removed normal medium from T75 culture flask and replaced with $CoCl_2 \cdot 6H_2O$ containing a medium. Cells were cultured 24 hours in a conventional incubator at 37 °Ca and 5% CO_2. Afterward, cells were harvested and prepared in the 96 well plates as it is mentioned in MTT assay protocols.[4,5]

Crystal violet assays: The medium was removed from the 96 well plate, then washed the plate with 100 µL PBS. 100 µL of one to one ratio mixed methanol and PBS was added cover the colonies and leave for 15 min. Subsequently, removed and added 100% methanol for a further 15 min fixation. Afterward, removed and added 0.5% crystal violet solution and left to allow sufficient staining for 20 min. Finally, removed the crystal violet solution and carefully rinsed with an indirect flow of water. Inverted and left to dry on the bench at room temperature. 200 µL of methanol was added to each well and left the plate with its lid on for 20 min at room temperature on a bench rocker with a frequency of 20 oscillations per minute. Read plates on a plate reader by using the light at 570 nm wavelength.[6,7,4]

7. UV-vis-NIR

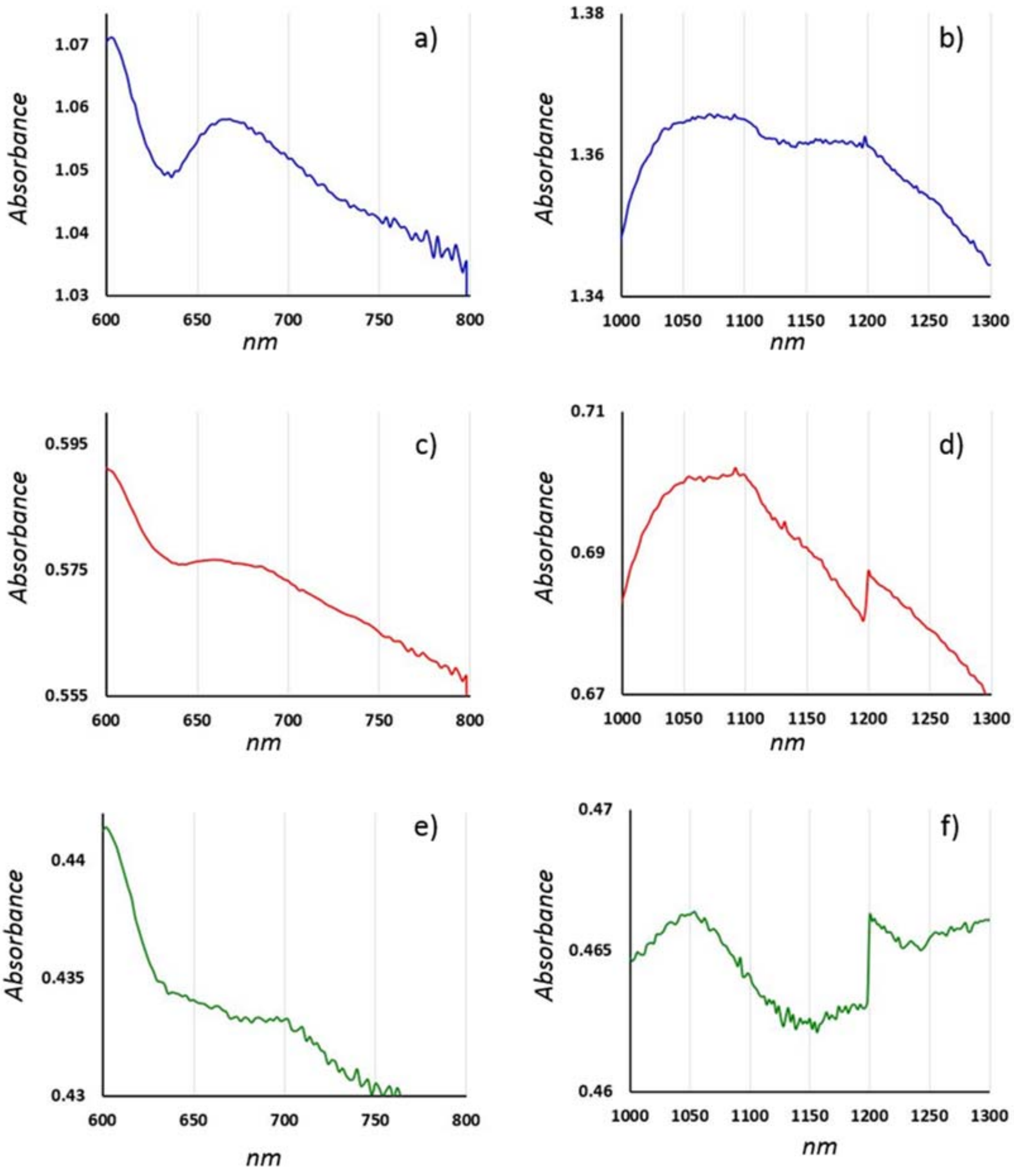

Figure S22. Optical absorbance spectra of CdSe@SWNTs@glucan blue lines), CdSe@SWNTs (Arc, red line) and CdSe@SWNTs (CVD, green line) 0.5 mg/mL dispersions in DMSO-d_6. Higher values of absorbance associated with the S_{11} (600-800 nm; a,c and d) and S_{22} (1000-1300 nm; b, d and f) interband transitions are seen for the DMSO-d_6 dispersion of CdSe@SWNTs@glucan, suggesting the adjuvant role of the β-D-glucan fibers in increasing SWNTs solubility.

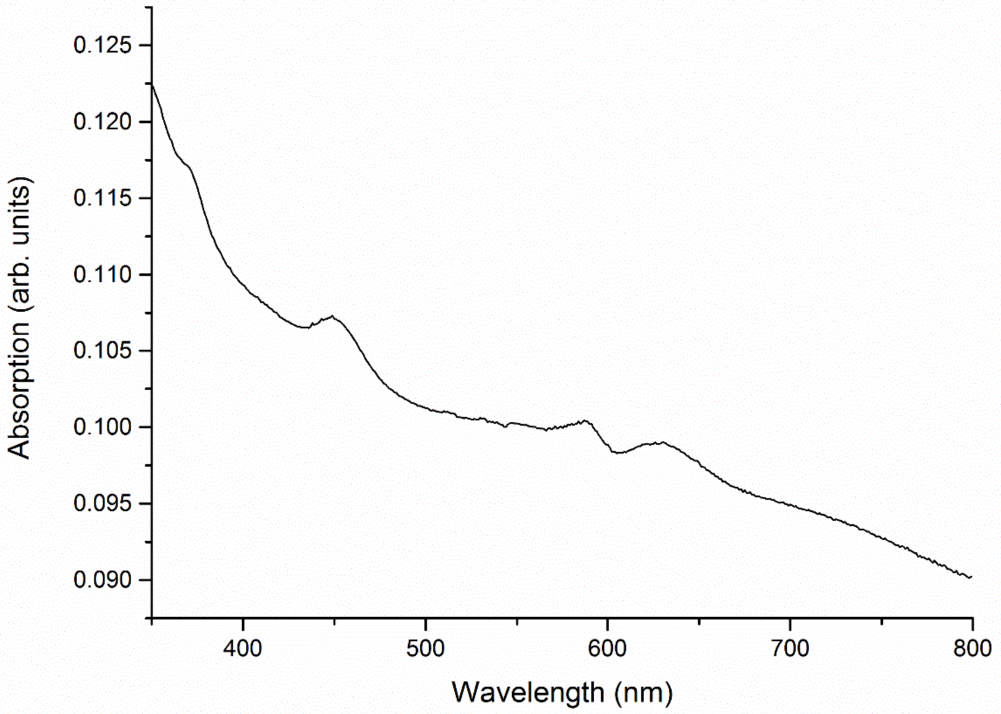

Figure S23. UV-vis optical absorbance spectra of CdSe quantum dots.

12. References

(1) Abou-Hamad, E.; Babaa, M. R.; Bouhrara, M.; Kim, Y.; Saih, Y.; Dennler, S.; Mauri, F.; Basset, J. M.; Goze-Bac, C.; Wågberg, T. Structural properties of carbon nanotubes derived from 13C NMR. *Phys. Rev. B,* **2011,** *84*, 165417-165417.

(2) Tyson, J. A.; Mirabello, V.; Calatayud, D. G.; Ge, H.; Kociok-Köhn, G.; Botchway, S. W.; Dan Pantoş, G.; Pascu, S. I. Thermally Reduced Graphene Oxide Nanohybrids of Chiral Functional Naphthalenediimides for Prostate Cancer Cells Bioimaging. *Adv. Funct. Mater.* **2016,** *26*, 5641-5657.

(3) Hu, Z.; Pantoş, G. D.; Kuganathan, N.; Arrowsmith, R. L.; Jacobs, R. M. J.; Kociok-Köhn, G.; O'Byrne, J.; Jurkschat, K.; Burgos, P.; Tyrrell, R. M.; Botchway, S. W.; Sanders, J. K. M.; Pascu, S. I. Interactions Between Amino Acid-Tagged Naphthalenediimide and Single Walled Carbon Nanotubes for the Design and Construction of New Bioimaging Probes. *Adv. Funct. Mater.* **2012,** *22*, 503-518.

(4) Vengellur, A.; LaPres, J. The role of hypoxia inducible factor 1α in cobalt chloride induced cell death in mouse embryonic fibroblasts. *Toxicol Sci.* **2004,** *82*, 638-646.
(5) Wu, D.; Yotnda, P. Induction and testing of hypoxia in cell culture. *J Vis Exp* **2011**, e2899-e2899.
(6) Alnuqaydan, A.; Sanderson, B. Toxicity and Genotoxicity of Beauty Products on Human Skin Cells. *J. Clin. Toxicol.* **2016,** *6*, 315.
(7) Chiba, K.; Kawakami, K.; Tohyama, K. Simultaneous evaluation of cell viability by neutral red, MTT and crystal violet staining assays of the same cells. *Toxicol. In Vitro* **1998,** *12*, 251-258.
